# Gold Nanoparticles: Construction for Drug Delivery and Application in Cancer Immunotherapy

**DOI:** 10.3390/pharmaceutics15071868

**Published:** 2023-07-02

**Authors:** Huiqun Huang, Ronghui Liu, Jie Yang, Jing Dai, Shuhao Fan, Jiang Pi, Yubo Wei, Xinrong Guo

**Affiliations:** 1 Guangdong Provincial Key Laboratory of Medical Molecular Diagnostics, The First Dongguan Affiliated Hospital, Guangdong Medical University, Dongguan 523808, China; huanghuiqun51@163.com; 2 Dongguan Key Laboratory of Environmental Medicine, School of Public Health, Guangdong Medical University, Dongguan 523808, China; Yang_J@gdmu.edu.cn (J.Y.); hila_l0229@163.com (J.D.); 3 School of Microelectronic, Southern University of Science and Technology, Shenzhen 518000, China; liurh@sustech.edu.cn; 4 Institute of Laboratory Medicine, School of Medical Technology, Guangdong Medical University, Dongguan 523808, China; fsh19854567217@163.com (S.F.); jiangpi@gdmu.edu.cn (J.P.); 5 Yunnan Key Laboratory of Pharmacology for Natural Products, School of Pharmaceutical Sciences, Kunming Medical University, Kunming 650500, China

**Keywords:** gold nanoparticles, drug delivery, cancer immunotherapy

## Abstract

Cancer immunotherapy is an innovative treatment strategy to enhance the ability of the immune system to recognize and eliminate cancer cells. However, dose limitations, low response rates, and adverse immune events pose significant challenges. To address these limitations, gold nanoparticles (AuNPs) have been explored as immunotherapeutic drug carriers owing to their stability, surface versatility, and excellent optical properties. This review provides an overview of the advanced synthesis routes for AuNPs and their utilization as drug carriers to improve precision therapies. The review also emphasises various aspects of AuNP-based immunotherapy, including drug loading, targeting strategies, and drug release mechanisms. The application of AuNPs combined with cancer immunotherapy and their therapeutic efficacy are briefly discussed. Overall, we aimed to provide a recent understanding of the advances, challenges, and prospects of AuNPs for anticancer applications.

## 1. Introduction

Cancer immunotherapy, which enhances the immune response and relieves immunosuppression, is a promising therapeutic prospect for cancer [1]. Compared with traditional treatments, such as chemotherapy, radiotherapy (RT), and surgery, immunotherapy offers distinct advantages, such as building long-term memory, preventing tumour recurrence, and controlling metastasis [2,3]. However, several challenges have impeded the widespread clinical application of cancer immunotherapies. One significant obstacle is the low clinical response rate, with less than 1/4 of patients showing a response to immune checkpoint inhibitors (ICIs) [4,5]. Although the combination of multiple ICIs can improve clinical efficiency, it also leads to serious adverse events, such as dermatitis and liver damage [6,7]. Therefore, ensuring the safety of immunotherapy is paramount. In recent years, nanoparticles have been used in drug design to improve the efficiency of drug-targeting delivery and enhance immunotherapeutic efficacy [8]. Among these nanoparticles, gold nanoparticles (AuNPs) have attracted widespread attention in cancer therapy owing to their low toxicity, high stability, ease of cellular uptake, excellent optical properties, and versatile surface functionalities [9,10,11,12,13].

The utilisation of AuNPs in cancer immunotherapy involves two main strategies. Firstly, AuNPs can serve as drug delivery systems to enhance the targeted delivery and efficacy of immunotherapeutic drugs. They can facilitate drug enrichment in tumour tissues via the enhanced permeability and retention (EPR) effect [14,15]. Drugs can be directly bound to the surface of AuNPs via covalent or noncovalent interactions [16,17]. Additionally, the surface of AuNPs can be modified to facilitate drug encapsulation and delivery. Targeted drug delivery can be achieved by employing specific modifiers, such as antibodies, aptamers, carbohydrates, and other ligands that recognise tumour-associated markers [18]. Some modifiers respond to external stimuli, such as pH or enzymes, enabling them to control drug release [19]. Secondly, the efficient photothermal conversion capability of AuNPs can be exploited to induce temperature changes for in situ drug release and tumour ablation. This process not only causes tumour immunogenic cell death (ICD) but also triggers innate or adaptive immune responses, thereby enhancing the anti-tumour immune response [20,21].

This review provides an in-depth analysis of the primary design strategies and delivery mechanisms employed in AuNPs loaded with immunotherapeutic agents or genes. Moreover, it highlights the process of drug dissociation from AuNPs to target tumour cells in detail. Finally, recent advances in the application of this material in cancer immunotherapy are discussed. We hope that this knowledge will facilitate the development of coordinated immune preparations to modulate the tumour immune response and enable a multi-platform treatment model.

## 2. Synthesis of AuNPs

The synthesis methods of AuNPs are ‘top-down’ and ‘bottom-up’. Among these, the ‘top-down’ method involves the physical decomposition of bulk gold material in a gaseous or liquid environment, utilising techniques such as laser ablation, sputtering, milling, thermolysis, ultraviolet or infrared irradiation, and vapour deposition [19,22,23,24,25,26]. This method typically yields highly pure AuNPs with uniform dispersion. However, this synthesis method not only requires more energy but also has higher instrumentation requirements. Additionally, defects in the surface structure of the products significantly impact their physicochemical properties [27,28,29]. The ‘bottom-up’ method usually involves the self-assembly of gold atoms or molecules into uniformly sized AuNPs, which is cost-effective and easily controlled [30]. This method includes chemical synthesis (spinning, sol-gel process, and chemical reduction), biosynthesis, and physical hybridisation methods (electrochemical, sonochemical, and photochemical synthesis) [31,32]. The first two chemical synthesis methods are mainly used for the synthesis of nanofibres and nanogels, which are mostly used for the production of industrial materials [33]. Among these methods, the chemical reduction method stands out because of its advantages of homogeneity, dispersion, enhanced stability, and precise control over size and shape. Approximately 60% of the AuNPs reported in the biological field were prepared using these methods from 2013 to 2018 [34]. Biosynthesis, an economical, environmentally friendly, and highly biocompatible approach, has gained popularity among researchers in recent years [35]. Furthermore, physical hybridisation methods have also been adopted for the synthesis of AuNPs in electrochemical or biosensor applications [36,37]. Table 1 shows a detailed description of the chemical reduction (Turkevich method, Brust–Schiffrin method, and Seeding-growth method) and biosynthesis methods.

### 2.1. Chemical Reduction Method for Synthesis of AuNPs

**Figure 1:**
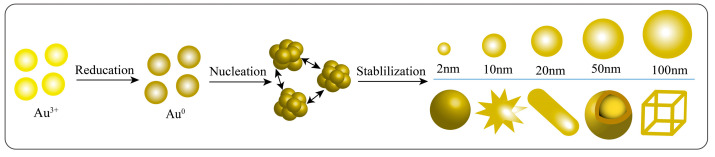
AuNPs with different sizes and morphologies were synthesized by the chemical reduction.

The chemical reduction method involves two main steps in the synthesis of AuNPs. Firstly, the reducing agent provides electrons to rapidly reduce Au^3+^ to Au^0^, forming small clusters (Figure 1) [55,56]. The reducing agents mainly include AA, borohydrides, citric acid, and hydrogen peroxide [34]. Secondly, stabilising/capping agents control the growth rate, final size, and morphology of AuNPs. These agents mainly include trisodium citrate dihydrate, surfactants (especially CTAB), and sulphur ligands [57]. In some cases, the reducing agent and stabilising/capping agent can be the same substance [30,58]. According to the differences in the synthesis system and conditions, this procedure can be classified into the Turkevich method, Brust–Schiffrin method, or seeding-growth method.

The classical Turkevich method was first proposed by Turkevich in 1951 and refined by Frenc in 1973. This approach produces spherical AuNPs in a homogeneous aqueous solution system in which trisodium citrate serves as both a reducing and stabilising agent [59,60]. In 2015, Polte elaborated on the theory of AuNP formation in detail. (1) Au^3+^ was rapidly reduced to Au^0^ and aggregated to form 1–2 nm clusters, and the decrease in the reduction rate resulted in the continued aggregation of unstable Au^0^. (2) The size of the clusters reached 2.5 nm, their numbers remained stable, but their sizes became larger and larger because of the poor dispersion of Au^0^. (3) After the size reached 4.5 nm, the reduction rate accelerated and consumed the remaining Au^3+^ in the solution. The reaction was terminated when the colour of the solution changed to ruby red [61,62]. Typically, this method synthesises mainly spherical AuNPs with a particle size distribution between 10 and 30 nm under heating. The resulting AuNPs exhibit excellent dispersion and low toxicity. Based on the classical Turkevich method, Netti et al. synthesised AuNPs with different morphologies, such as nanowires, nanoflowers, nanowebs, and nanoplates at room temperature. The formation of these morphologies was achieved via the cold fusion of ultra-small spherical AuNPs [63]. Furthermore, other substances, including L-tyrosine [64,65], ethanol [66], and poly-vinyl-pyrrolidone [67], were added to the reaction to manipulate the morphology and size of AuNPs at room temperature. To analyse the aggregation mechanism of AuNPs, the authors also developed a numerical diffusion-limited aggregation model to simulate the dimensions and growth kinetics of AuNPs [68]. This report provides insights into the mechanism of reduction in gold ions by citric acid molecules. However, the Turkevich method has some shortcomings. For instance, the product yield is low, and nanoclusters aggregate uncontrollably, leading to irregular nanoparticles greater than 30 nm in size [30,55].

The Brust–Schiffrin method, based on the biphasic liquid–liquid system, overcomes the problem of severe agglomeration encountered in inhomogeneous systems [69]. Commonly, TOAB is used as a two-phase transformer to transfer the HAuCl_4_ solution into the organic phase. Subsequently, reducing agents, such as CTAB and sodium borate, are employed to reduce gold ions and form AuNPs with a thiol layer [56,70]. The thiol layer has a strong affinity for inhibiting the growth of AuNPs, resulting in small nanoparticles. This method allows the preparation of spherical AuNPs with excellent stability and a controlled particle size of <10 nm [34]. However, poor dispersion of the prepared AuNPs is the largest challenge [19,71].

These two methods are mainly used for the synthesis of spherical AuNPs, whereas the synthesis of rod or heterogeneous AuNPs suffers from low yields and product defects [72]. The Seeding-growth method addresses these issues. The method consists of two main steps. (1) A strong reducing agent (NaBH_4_) is used to reduce HAuCl_4_ to form seeds. (2) The seed solution is added to a gold salt solution containing a weak reducing agent (e.g., AA), followed by the addition of a molecule (e.g., CTAB) to induce the growth of the seeds into Au NRs [73]. To achieve better control of the shape and increase the yield of the nanorods, a low concentration of additional ions, such as metal ions and halides, is added before the growth phase [74,75]. However, size and shape are influenced by various factors, such as seed concentration and size, reductant concentration, pH, temperature, and even the solution order [76].

### 2.2. Biosynthesis of AuNPs

Eco-friendly and non-toxic biosynthesis has also been widely explored for the synthesis of AuNPs, considering the toxicity and occurrence of adverse reactions in chemical methods [32,77,78]. Many organisms, including plants and microorganisms (e.g., bacteria, fungi, algae, and yeast), have been demonstrated to act as reducing agents or synthesis sites for the preparation of AuNPs [79,80]. The mechanism of this process involves the reduction of Au^3+^ to Au^0^ using biomass. Biomass contains a variety of organic compounds including phenols, terpenes, amino acids, alkaloids, steroids, coenzymes, flavonoids, proteins, reducing sugars, nicotinamide adenine dinucleotide, and water-soluble alkaloids [81,82,83,84].

Plants are the most popular raw materials for biosynthesis as they contain large amounts of reducing substances and are cheap and readily available. The first successful synthesis of AuNPs using biomass was reported in 1999 [85]. Since then, each part of the plant, including leaves, bark, flowers, fruits, seeds, rhizomes, and bark, has been used as a raw material to prepare AuNPs [86]. Usually, leaves and fruits are especially popular as they contain high concentrations of reducing substances [28]. This method enables the synthesis of AuNPs with different shapes, structures, and physicochemical properties, with size distributions mainly in the range of 5 to 400 nm [87,88]. Plants are suitable for the large-scale production of Au NPs because they do not require a sterile environment or complex processing. However, the effect of plants on AuNPs has not been elucidated because of their complex composition [86,89,90].

Some microorganisms can also synthesise AuNPs by enzymatic reactions that occur via extracellular biosorption, biomineralisation, intracellular bioaccumulation, and metabolic processes. These enzymes enable the reduction of Au^3+^ to Au^0^ [91]. The shape, size, dispersion properties, and stability of nanoparticles can be controlled by adjusting the external conditions [92]. Among them, the preparation of AuNPs using bacteria includes three main synthesis mechanisms. (1) Enzymes produced by bacteria, such as *Rhodopseudomonas capsulatus*, *Thermomonospora* sp., *Pseudomonas denitrificans*, *Shewanella algae*, *Deinococcus radiodurans*, *Rhodococcus*, *Bacillus megaterium*, *Bacillus subtilis*, and *Escherichia coli*, have been used to reduce Au^3+^ to AuNPs. These enzymes are produced by metal adsorption and metabolism in bacteria [80,93,94,95,96,97,98]. (2) Bacteria produce active secretions with reducing properties. For example, nicotinamide adenine dinucleotide hydride (NADH)-dependent reductase plays a major role in the reduction in Au^3+^ to AuNPs via electron transfer from NADH [99,100]. (3) Other active substances in bacteria, such as cell wall reductive enzymes, carotenoids, and cysteine desulfhydrase, can also synthesise AuNPs [101,102]. These enzymes and organic substances can be used simultaneously as capping agents to stabilise and prevent the agglomeration of AuNPs. Furthermore, bacterial synthesis is an exact and slow procedure that requires optimisation of the synthesis state. Fungi are easier to culture and grow than bacteria. They are also able to secrete a number of protein-reducing substances for the synthesis of AuNPs, such as the 10-kDa protein found in *Fusarium oxysporum*, 80–10 kDa protein found in *Thermomonospora* sp., glutathiones, and α-NADPH-dependent sulphite reductase [103,104]. Some fungi are potentially pathogenic [105,106]. The metabolic environment of microorganisms is crucial for the synthesis of AuNPs and therefore needs to be optimised for their synthesis. However, the intracellular environment cannot be observed and regulated in real-time, which can affect the synthesis of AuNPs [91,92,107].

AuNPs have extensive applications in cancer treatment and drug delivery. Owing to their unique physicochemical properties, including a high X-ray absorption coefficient, photosensitisation, surface plasmon resonance (SPR), ease of functionalisation, and stability. The optical and physical properties of AuNPs are dependent on their size, shape, and surface chemistry, which in turn affect their drug-carrying capacity, organ distribution, pharmacokinetics, photothermal conversion efficiency, and immunological properties as drug carriers. Therefore, it is important to thoroughly evaluate the properties of AuNPs prior to constructing drug carriers [108,109,110]. Various sophisticated techniques are available for characterising the size, shape, and chemical properties of AuNPs. Ultraviolet-visible spectroscopy is commonly employed to characterise the SPR of AuNPs, providing a preliminary analysis of AuNP size and distribution and assessing AuNP aggregation. Transmission electron microscopy reliably measures AuNP size and provides a clear structural morphology [111]. Dynamic light scattering is useful for testing aggregation, and measuring the zeta site of AuNPs can determine their surface charge and solution stability. To investigate surface-bound substances and their forms, X-ray photoelectron spectroscopy and Fourier transform infrared spectroscopy can be used to analyse the surface chemical structures of AuNP complexes [112]. Moreover, the quantity of drugs bound to the AuNPs can be calculated using ultraviolet detection technology [113].

In conclusion, there are various methods to prepare AuNPs, all of which have advantages and disadvantages. When AuNPs are used as drug carriers, various factors must be considered, such as the effects of size, shape, and surface properties on drug loading, in vivo transport efficiency, biocompatibility, and immune response. Next, we detail the use of AuNPs as drug carriers and their application in cancer immunotherapy.

## 3. AuNPs-Mediated Drug Delivery

Drug delivery through carriers is a fascinating and promising tactic to enhance therapeutic effects. This facilitates the release of the drug at the desired time and space, enhancing the efficiency of the drug and minimising its systemic toxicity. AuNPs have also been developed as promising drug carriers for binding different types of drugs and target ligands. Controlled drug release is achieved by surface modification of the design on AuNPs.

### 3.1. Target Drug Loading

Drug loading refers to modification or loading of the original drug before administration, primarily to improve its stability and reduce its side effects. AuNPs bind drugs using two strategies: covalent and non-covalent binding (Table 2). Non-covalent binding mainly involves electrostatic modification, van der Waals interactions, π-π stacking, and hydrophobic effects [19,114,115]. However, it exhibits poor selectivity and weak adsorption strength. The weak adsorption leads to easy separation of drugs from AuNPs under the influence of the external environment. For example, the electrostatic adsorption of AuNPs is easily affected by pH, and hydrogen bonds are easily interrupted by polar molecules (e.g., water) [116]. This leads to premature drug release and a sudden increase in plasma concentration, which then produces toxic side effects and accelerates drug metabolism [117]. In contrast, covalent bonds formed through Au-S and Au-N are more stable to avoid sudden or rapid drug release, achieving controlled drug release [118,119,120].

To enhance drug binding with AuNPs, intermediate linkers have been introduced because not all drugs can be directly connected to AuNPs. Several researchers have observed an effective increase in drug affinity with AuNPs using PEG-modified compounds to provide more binding sites for drugs. For example, doxorubicin (DOX) and anti-PD-L1 form a stable drug delivery system with AuNPs by amide binding to lipoic acid polyethylene glycol N-hydroxysuccinimide (LA-PEG-NHS). The anti-PD-L1 antibody reacts with a 5-fold molar excess of LA-PEG-NHS, and its binding affinity is greater than 90% [130]. However, when hydrophobic drugs linked to PEG are exposed on the surface of AuNPs, drug solubility, dispersion, plasma concentration, and circulation time are reduced. To address these issues, Cui et al. developed an AuNP@Dox-mPEG system by modifying the position of PEG to encapsulate DOX. This modification significantly improves the plasma half-life and stability of the drug [16]. Furthermore, encapsulating drugs using dendrimers and liposomes can help reduce premature drug leakage and adverse reactions [131]. The drug encapsulation rate increased to 86.5% and the leakage rate decreased from 47.5% to 7.8% within 12 h after using the cell membrane to encapsulate drugs at pH 7.4 [132].

### 3.2. Delivery of AuNPs to Tumour Tissue

**Figure 2:**
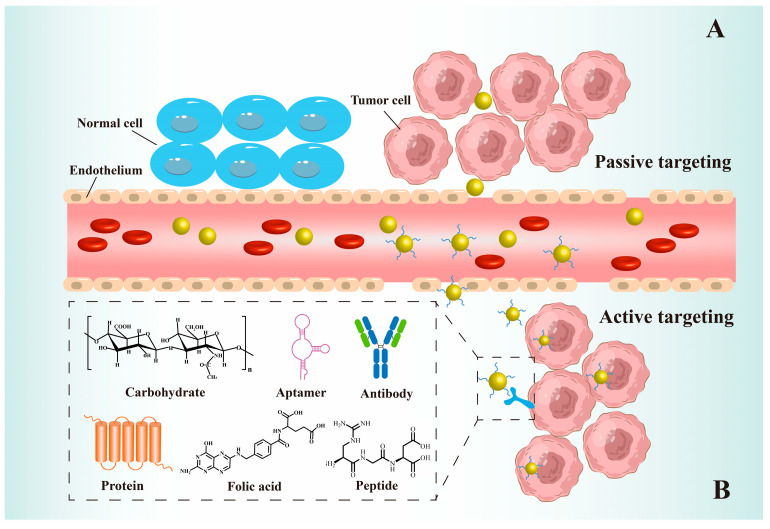
Targeting mechanisms based on delivery of AuNPs. (**A**) Passive targeting uses the effect of leaking tumour vasculature-mediated EPR to passively deliver AuNPs-loaded therapeutic agents into the tumour. (**B**) Active targeting refers to the specific binding of AuNPs to receptors on the surface of tumour cells through the functionalisation of targeting ligands.

AuNPs coupled with targeted molecules can accurately deliver tumour-targeting drugs through both passive and active targeting (Figure 2) [133,134]. In passive targeting, AuNPs mainly exploit the EPR effect. Owing to vascular leakage and impaired lymphatic drainage, drugs attached to AuNPs can preferentially accumulate in tumour tissues and remain there for a long time [135,136,137]. The EPR effect is influenced by the physical properties of the AuNP carriers, such as size, shape, and surface charge. These factors affect the localisation, cell penetration, and load release rate of carriers. For example, nanoparticles below 50 nm have the highest aggregation in tumours owing to their highly organised and osmotically pressurised environment [138]. Nanoparticles smaller than 10 nm are rapidly cleared by the kidneys, whereas those larger than 150 nm can be easily removed by the reticuloendothelial system and undergo nonspecific aggregation in the liver and spleen [139,140,141,142]. Additionally, surface chemistry and charge strongly influence the cycle time of AuNPs, with superhydrophobic or charged systems being susceptible to mononuclear phagocyte systems [143,144]. Designing hydrophilic, neutral, or slightly anionic drug delivery systems not only avoids the above-mentioned effects but also enables precise drug delivery using the EPR effect [145]. However, to date, nanoparticle-based chemotherapeutic agents have failed in clinical trials [146,147,148]. According to a meta-analysis of 117 nanodrug delivery studies, only 0.7% of nanoparticles reach the tumour site [149]. This suggests that although passive targeting enhances the effective deposition of AuNPs in the tumour mesenchyme, it is insufficient for promoting the localisation and uptake of AuNPs in tumour cells.

Drug-loaded AuNPs can enable active tumour targeting via antibody-modified ligand strategies [18,130,150]. The most commonly used antibody-targeted molecules include immunoglobulins and antibody fragments [18]. For example, AuNP-modified anti-programmed cell death protein 1 (PD-1) antibodies can effectively target colorectal cancer cells [130]. The delivery system can alter the pharmacokinetics and uptake of drugs [18]. Nonetheless, the high antibody-target affinity depends on non-functional and barrier-free antigen-binding sites. Furthermore, the strategy of binding antibodies to amide bonds forms active sites oriented toward the gold core, reducing the binding capacity of the antibody [151]. To identify the most optimal antibody and develop a suitable linkage strategy, further research is needed to investigate the pharmacokinetics of linked antibodies.

Several types of aptamers coupled with AuNPs have been developed for active targeting, including nucleolin-targeting aptamer-AS1411, protein tyrosine kinase 7 (PTK7) selective aptamer-Sgc8c [152], prostate-specific membrane antigen aptamers [153], and aptamer MUC1 (a transmembrane protein) [154]. Aptamers have been considered an alternative to antibodies owing to their chemical synthesisability and ease of modification [155]. In one report, MUC1-modified PEG-AuNPs successfully delivered paclitaxel precisely to breast cancer cells [156]. The specificity of MUC1 for surface receptors of breast cancer cells rendered AuNP-mediated drug delivery 96% more efficient. Another innovative drug delivery system was achieved using a multivalent aptamer system containing AS141 and Sgc8c bound to AuNPs. This system enabled AuNPs loaded with chemotherapeutic drugs to exhibit a high degree of specificity for cancer cells expressing nuclear phosphate and PTK7 [152].

Many peptides, such as integrin ligand cyclic peptide C, vasoactive peptides, neuropeptides, and cellular membrane-penetrating peptides, have been utilised for targeted delivery of therapeutic AuNPs [18]. Peptide-modified AuNPs are more suitable for nuclear targeting than antibodies and some proteins because of their low molecular weight. This property enables peptides to easily pass through the nucleus, resulting in efficient nuclear delivery [157]. The use of nuclear-targeted peptides guides functionalised AuNPs for targeted cell internalisation and efficient nuclear-targeted transport in breast cancer cells [158]. Moreover, Paulo et al. revealed that the utilisation of peptide-modified AuNPs increases both drug accumulation and half-life, ultimately leading to a 50% improvement in radionuclide uptake by human glioblastoma cells [159]. This effect is due to the ability of AuNPs to reduce peptide interactions with serum proteins, enhance peptide stability, and prolong peptide retention in tumour cells (up to 72 h).

The specificity of proteins can facilitate the active targeting of the AuNP drug delivery system. Lee et al. constructed AuNPs bound to lactoferrin (Lf), a milk protein receptor, resulting in sustained, controlled, and targeted drug delivery to glioblastomas [160]. Compared with that of unmodified AuNPs, the oral administration of Lf-modified AuNPs to mice yielded an 8-fold increase in AuNP in the brain. Lectins can also be used in cancer-targeted therapy. David et al. demonstrated the first use of jacalin to bind to the amide bond of AuNPs, enabling them to target cells that express T antigens [161]. Fluorescence localisation revealed that jacalin-mediated AuNPs are uniformly distributed within the cell and significantly more endocytosed than unmodified AuNPs. The anti-tumour efficacy of Jacalin-mediated AuNPs is significantly enhanced, exhibiting approximately 10-fold greater potency than unmodified AuNPs.

Carbohydrates, natural targeting ligands, can target lectins that are abnormally highly expressed on the surface of cancer cells. For example, hyaluronic acid (HA) is commonly used to target tumours with the high expression of CD44 receptors, such as melanoma. Thiol groups can be introduced into HA and used to covalently link AuNPs via Au-S for the treatment of cutaneous melanoma. Quantitative analysis of fluorescence intensity revealed a 2.23-fold increase in the uptake rate of HA-AuNPs compared with free HA molecules [162]. However, some carbohydrates have an affinity for multiple receptors, which may reduce targeting and lead to off-target effects [163].

Not all tumour cells stably overexpress receptors; thus, the surface density of targeted ligands has an important effect on the specific targeting of cells by nanocarriers. Generally, a higher density of ligands within a certain range results in more efficient and precise targeting [133]. Cancer immunotherapy targets are under investigation. Only a few targets have been identified, such as immune checkpoints, which are influenced by cancer cell differentiation. However, some targets are underexpressed or blocked in certain cancer cells, necessitating careful consideration of these issues and drug resistance in practical applications. Therefore, it is crucial to identify new targets and provide innovative therapeutic options.

### 3.3. Drug Dislocation from AuNPs

**Figure 3:**
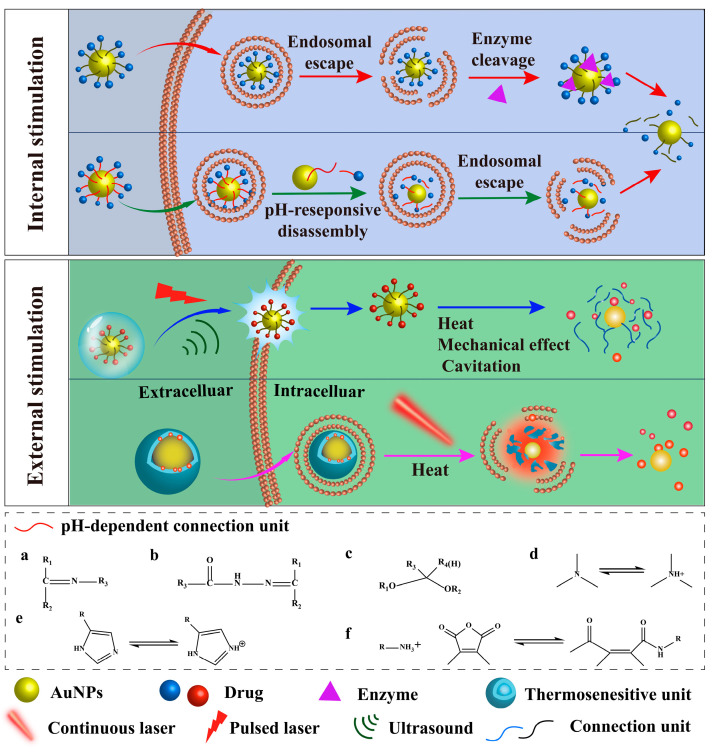
Strategies of drug release mediated by AuNPs as carriers primarily include internal and external stimulation. The internal stimulation-mediated drug release mainly includes (I) enzyme-stimulated and (II) pH-responsive drug release. The PH-responsive drug release is mediated by pH-sensitive connection units, commonly including (**a**) Schiff base compounds, (**b**) acyl-brown bonds [168,169], (**c**) acetal/ketone structures [170], (**d**) tertiary amino protonation [171], (**e**) the reaction of amino groups with 2,3-dimethylmaleic anhydride [172], and (**f**) the protonation of imidazole groups [173]. The external stimulation is based on the ultrasound, pulsed laser, and continuous laser external stimulation of AuNPs.

The mechanism of AuNP-loaded drug release within a tumour can be classified into internal and external stimulation based on the source of stimulus release (Figure 3). Internal stimulation strategies are based on differences between tumour and normal tissue environments, such as pH and enzymes. pH-sensitive site-specific drug release is induced by the acidic tumour microenvironment (TME) [164,165]. Under specific pH conditions, the pH-sensitive connection between the drug and AuNPs undergoes cleavage owing to carrier protonation and morphological changes, leading to drug release. The commonly used pH-sensitive connection units are shown in Figure 3a–f. For example, the introduction of pH-sensitive Schiff base bonds between AuNP-labelled antigens can lead to an approximately 20-fold increase in antigen release in an acidic environment [166]. Additionally, some pH-sensitive polymers have been used for the internal stimulation of drug release. For example, by covalently binding S-nitrosoglutathione (GSNO) to AuNPs, the pH-sensitive cationic polymer PAA enables the release of 19.8 ± 3.0% of GSNO at pH 7.4 over a period of 72 h. Under weakly acidic conditions (pH 6.5 and 5.0), the compatible GSNO release rates were measured as 60.5 ± 2.5% and 87.6 ± 2.0%, respectively. This increase was due to the opening of the PAA structure upon acidification [125]. Notably, this approach is only applicable to acidic tumours and has limitations under alkaline or physiological conditions [167].

Enzyme reactions are a promising smart stimulation approach, mainly using tumour-specific highly expressed enzymes to trigger drug release, such as matrix metalloproteinase (MMP) and hyaluronidase (HAase). MMP-2, a member of the MMP family, is highly expressed in breast cancer [174]. An MMP-2 sensitive peptide, R9, has been introduced between an immunostimulatory molecule (TLR7/8a imidazoquinolone) and AuNPs to prepare breast cancer vaccines [175]. R9 facilitates endocytosis and antigen capture in AuNP delivery systems. Moreover, R9 can be cleaved at high concentrations of MMP-2 in tumour cells to separate TLR7/8a imidazoquinoline from AuNPs. This exposes the TLR7/8a imidazoquinoline, which activates the TLR7/8 pathway and enhances the stimulation of host dendritic cells (DCs). Consequently, this approach amplifies adaptive anti-tumour T cell responses, triggers effector memory immune responses, and activates innate anti-tumour immunity. HAase is another enzyme highly expressed in breast cancer that cleaves the β-1-4-glycosidic bond in HA, leading to specific drug release within breast cancer cells [176]. Modification of HA on AuNPs loaded with DOX not only targets cancer cells that overexpress the CD44 receptor but also prevents premature drug release. In the presence of HAase, DOX release increases from 5.6% to 49% within 48 h [177]. Nonetheless, the efficacy of enzyme-triggered drug delivery is contingent on the specificity and activity of the enzyme, and constructing carriers for this approach remains challenging owing to the complicated in vivo enzyme environment.

External stimulation methods based on the local surface plasmon resonance of AuNPs have been developed, such as photothermal, laser optoporation, and ultrasound. The photothermal strategy involves incorporating a thermosensitive unit between the AuNPs and drug, where temperature changes act as stimuli for drug release. Chae et al. found that the rate of drug release depends on the binding force of the thermal unit and AuNPs [178]. Their study showed that the terminal sulfhydryl groups of mercaptosuccinic acid (MSA) result in a nearly 2-fold reduction in drug release under the same laser excitation conditions compared with mercaptopropionic acid. This was due to the stronger binding affinity between MSA and AuNPs. However, this method of external attachment suffers from premature rupture and decomposition during in vivo transport. To address this issue, Pourjavadi et al. employed thermosensitive micelles to wrap AuNPs and paclitaxel to control drug release [179]. They used a near-infrared (NIR) laser to stimulate the AuNPs, resulting in a 50% increase in effective drug release. Additionally, this method increases the skin penetration depth while reducing potential tissue damage. This is because the NIR laser has a penetration depth of more than 1 cm, a spatial resolution of tens of microns, and an efficiency of more than 1000-fold compared with visible light [180]. However, the continuous laser used in this method typically requires more than the maximum permissible exposure (MPE) of the laser to achieve the desired treatment effect (1 and 0.33 W cm^−2^ skin-to-laser MPE) [181]. Therefore, damage to normal tissues caused by tissue self-absorption cannot be avoided.

Compared with those with continuous lasers, pulsed lasers reduce the required dose of AuNPs, laser power threshold, and extent of thermal damage. The mechanism of this method involves AuNPs absorbing pulsed laser light, resulting in the rapid evaporation of water vapour to form micro/nanobubbles. These micro/nanobubbles can then suddenly rupture due to continuous warming, generating mechanical forces that create pores in the cell membrane, facilitating intracellular delivery of biomolecules [182,183]. AuNPs absorbing pulsed laser-generated mechanical stress enhance the permeability of the nuclear membrane and improve the transfection efficiency of caspase9-ribonucleoproteins by approximately 55% [184]. Furthermore, by attaching AuNPs to T cells, optoporation increases the nuclear transfection rates of pericytes. Subsequent downregulation of immunosuppressive pathways significantly enhances the ability of T cells to infiltrate tumours and eliminate cancerous cells [185]. The resulting cell permeability is correlated with the power and wavelength of the laser. At low power, thermal effects dominate, whereas at high power, mechanical effects are more prevalent [186]. Yao et al. demonstrated that shorter-wavelength lasers (530 nm) have higher penetration rates, whereas longer-wavelength lasers (730 nm) penetrate deeper into tissues and enhance drug cellular internalisation by up to 50% [187]. This is due to the self-limiting effect of transient or permanent absorption. Moreover, the NIR-II region (900–1700 nm) laser has deeper tissue penetration and less tissue scattering than the NIR-I region (650–900 nm) [188]. Bibikova et al. explored the effects of different pulsed laser wavelengths on cell permeability by evaluating fluorescent dyes. Their findings demonstrated that the cell permeability of the 1064 nm laser-mediated dye increases nearly 20-fold compared with that of the 650 nm laser [189]. Under the same laser, Pylaev et al. additionally noted that the geometry of AuNPs correlates with cell membrane permeability under a 1064 nm pulsed laser. The most substantial cell penetration and survival rates are observed with rod-shaped AuNPs [190]. Despite the potential benefits of laser treatment, it is prohibitively expensive, and the distribution of the produced micro/nanobubbles is not uniform. Consequently, the mechanical forces generated by bubble collapse can cause irreversible damage to the cell membrane. This is beneficial for killing cancer cells; however, safety issues should be fully considered.

Ultrasound is another method of external stimulation, the mechanism of which is thermal and mechanical effects and cavitation on AuNPs. AuNPs can provide nucleation sites for cavitating bubbles, reducing the ultrasound intensity required for cavitation and increasing the number of bubbles that collapse. Ultrasound can achieve greater penetration than lasers, enabling the formation of more uniform and stable micro/nanobubbles [191]. Tavakkoli et al. found that ultrasound-mediated drug release from curcumin-loaded AuNPs increases by 85% exclusively under mechanical action [192,193]. Researchers have found that mechanical effects and cavitation cause milder drug release, whereas high temperatures lead to drug denaturation and cell membrane collapse. Ultrasound pulses have also been shown to enhance cell permeability. Lin et al. employed AuNPs to deliver gemcitabine (Gem) and microRNA-21 inhibitors into the nucleus, resulting in an 82-fold higher gene inhibition than free Gem over 72 h [194]. Moreover, nanobubble rupture-induced mechanical stress can increase the permeability of the blood–brain barrier by inducing mechanical stress on the vessel wall. Using focused ultrasound in a non-invasive and targeted manner, Tiffany G. successfully delivered DNA-modified Au NPs to specific locations in the mouse brain for the first time. They also compared the penetration efficiency of different sizes of AuNPs and found that the smallest AuNPs tested in the study (6 nm) are delivered six times more efficiently in the blood–brain barrier than the largest AuNPs tested (14 nm) [195]. This contributes to the treatment of brain tumours. However, the non-specific and uncontrollable adsorption of AuNPs on membranes leads to non-targeted cell damage. Ultrasound has adverse cavitation effects in organs containing gas, such as the lungs and intestines, rendering them unsuitable for ultrasound treatment. Furthermore, the required exposure time and ultrasound intensity for drug release should be thoroughly considered.

To overcome the limitations of individual stimulation strategies, multiple combined stimulation strategies have been developed, the most commonly used being pH/NIR radiation dual stimulation. Chen et al. utilised a dual-sensitive platform to control the release of IDO inhibitors to activate T cells and improve immunotherapy effectiveness [125]. The platform was formed by the self-assembly of pH-sensitive PAA and temperature-sensitive β-β-titanium nitride on AuNPs. Under weakly acidic conditions, the PAA structure opened, resulting in a nearly 2-fold increase in IDO inhibitor release. When combined with NIR irradiation, drug release was further increased by approximately 15%. Consequently, the slow drug release from the pH pathway is overcome by the thermosensitive release pathway. The dual-sensitivity platform enabled flexible control of the rate and amount of drug release. AuNPs can also be combined with other nanoparticles to construct multiple-response delivery systems. The use of self-assembling AuNPs, hollow hydroxyapatite, and dopamine to form a pH/NIR dual-sensitive platform increased the release rate of DOX by a factor of 3.5 [196]. The increased release rate was attributed to the protonation of the amine group of dopamine in an acidic environment, disrupting the π-π interaction of DOX with dopamine. Furthermore, the thermal effect of the AuNPs disrupted the structure of the hollow hydroxyapatite.

## 4. Enhancement of AuNPs in the Immune Cycle

**Figure 4:**
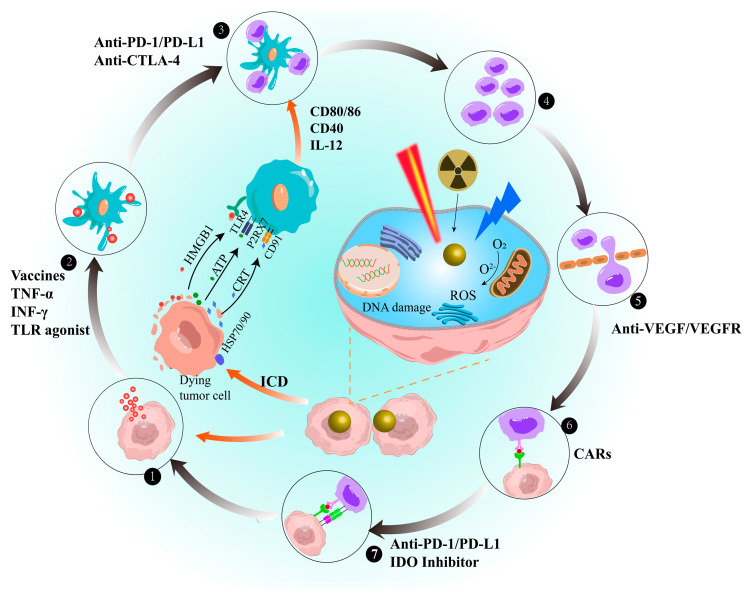
Enhancement of AuNPs in the cancer immunity cycle. CRT: surface-exposed calreticulin; ATP: adenosine triphosphate; HSP: heat shock protein; HMGB1: high mobility group box 1 protein; TNF-α: tumor necrosis factor-α; IL: interleukin; IFN-γ: interferon-γ; CTLA-4: cytotoxic T lymphocyte-associated antigen-4; CARs: chimeric antigen receptors.

Owing to the low immunogenicity of some tumours, the anti-tumour immune responses cannot be initiated [197]. However, AuNPs can enhance the immunogenicity of tumours, thereby increasing the ability of the immune system to recognise and kill tumour cells. AuNPs can act as carriers for immune agents to mediate the immune cycle and promote the release of damage signals from cancer cells, thereby recruiting immune cells to enter the cancer lesion to reduce tumour immune tolerance and enhance the immune cycle (Figure 4) [198,199].

**Figure 5:**
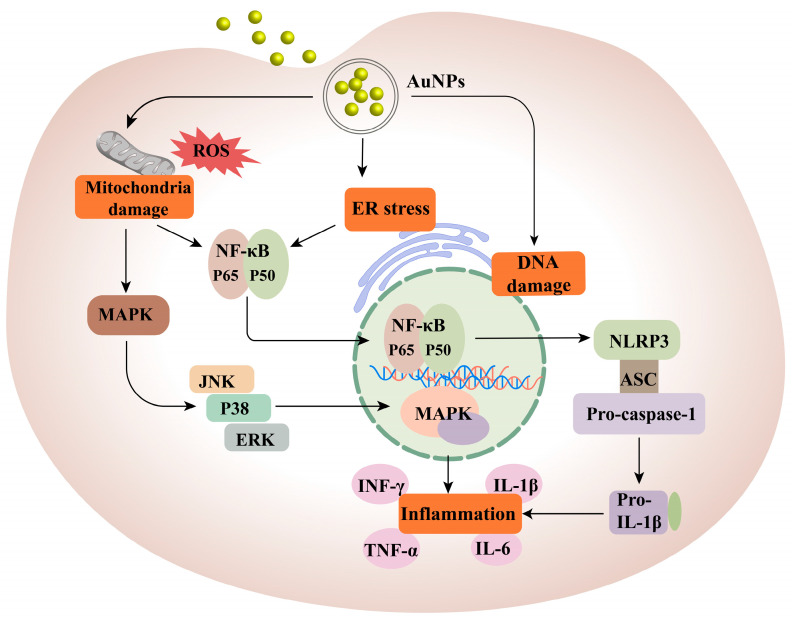
Mechanisms of intracellular AuNPs-mediated immune responses. NLRP3: NOD-like receptor thermal protein domain associated protein 3; ASC: apoptosis-associated speck-like protein containing a CARD; Pro-caspase-1:pro-cysteinyl aspartate specific proteinase-1; ER: endoplasmic reticulum; ROS: reactive oxygen species; MAPK: mitogen-activated protein kinase; NF-κB: nuclear factor kappa-B; ERK: extracellular regulated protein kinases; JNK: c-Jun N-terminal kinase.

AuNPs primarily strengthen the immune cycle via two pathways. The first pathway involves interactions with macrophages, DCs, T lymphocytes, and natural killer (NK) cells. This interaction causes oxidative stress, organelle dysfunction, and DNA damage reactions, which trigger different cell pathways, leading to apoptosis or inflammation. As a result, the ability of the immune system to kill cancer cells is enhanced [199]. Detailed descriptions of these two pathways are provided in Figure 5. AuNPs interacting with macrophages can activate redox-sensitive NF-κB and MAPK signalling pathways, promoting the release of pro-inflammatory cytokines (TNF-α and IL-1b) [200,201,202,203,204,205]. Proinflammatory cytokines stimulate specific T cell immune responses and promote macrophage differentiation. The effect of AuNPs on tumour-associated macrophages (TAM) favours the differentiation of M1 macrophages, which has an inhibitory effect on tumours [205]. Additionally, the reduction in regulatory T cell (Treg) and increase in NKT cell levels contribute to AuNPs inducing favourable pro-inflammatory and anti-TME responses [205]. Additionally, the reduction in Tregs and increase in NKT cells contribute to AuNPs inducing favourable pro-inflammatory and anti-TME effects.

Another approach is to induce ICD using AuNPs in RT, photothermal therapy (PTT), and photodynamic therapy (PDT) [21,206]. This method induces a favourable immune response at the tumour site, which transforms the immunosuppressive TME into an immunostimulatory TME. Consequently, it induces anti-tumour immune responses and enhances the therapeutic effect of immunotherapy. Studies have shown that AuNPs induce ER stress and ROS, leading to the activation of danger signal pathways in tumour cells. This activation promotes the secretion and release of damage-associated molecular patterns (DAMPs), such as CRT, ATP, HSP, and HMGB1 [207,208,209]. DAMPs can facilitate DC maturation, and mature DCs can secrete inflammatory factors (IL-12 and IFN-γ) and express co-stimulatory signals (CD80 and CD86) crucial for T cell differentiation [210]. Moreover, AuNPs in PDT can induce neutrophil aggregation at the lesion site within minutes by destroying subcellular structures and plasma membranes, thereby triggering inflammation. Neutrophils release lytic enzymes that destroy tumour cells and induce monocyte and macrophage infiltration [211,212]. This promotes the secretion of inflammatory cytokines and chemokines. Furthermore, the photothermal effect and ROS toxicity induced by AuNPs lead to cell death and the destruction of tumour blood vessels. This process causes the accumulation of AuNPs and continuous accumulation of dying tumour cells and DAMPs, which can promote the recruitment of more immune cells to infiltrate the tumour [213,214].

AuNPs can induce both pro-inflammatory and anti-inflammatory immune responses. This effect depends mainly on their physical and chemical properties, such as size, charge, shape, and functional groups. However, variations in the uptake dosage, method, and intracellular dynamics of AuNPs by the mononuclear-phagocyte system make understanding the mechanisms of immune responses challenging [215,216]. Therefore, achieving a balance between the interactions of AuNPs with different cells and inflammatory factors is a critical issue for the immunotherapeutic use of AuNPs.

In summary, AuNPs are promising potential carriers for cancer immunotherapy as they can enhance the immunogenicity of cancer cells and reduce the severe side effects of anticancer treatments. AuNP-triggered ICDs can increase the responsiveness of cancer immunotherapy. However, cancer cells often develop resistance to apoptosis; therefore, new strategies are needed to induce other forms of cancer cell death. Currently, combining treatment modalities with external stimuli appears to be a promising strategy.

## 5. Application in Cancer Immunotherapy

Cancer immunotherapy is a treatment method that overcomes the recurrence and metastasis of cancer cells by stimulating tumour-specific immune responses and immune memory [217,218]. AuNPs have been combined with RT, PTT, and PDT to enhance the anti-tumour effect and applied to animal models of various solid tumours. This application not only improves the anti-tumour effect but also reduces the recurrence/metastasis rate of tumours (Table 3).

### 5.1. AuNPs as Drug Delivery

#### 5.1.1. Cytokines

As cytokines have a short half-life, they must be administered at high doses, which can lead to serious side effects and reduce the effectiveness of cancer immunotherapy [230]. To address this, AuNPs have been utilised as vectors for cytokines, such as IFN, IL, and TGF-β. Gasparri et al. found that utilising AuNPs as carriers reduces the amount of IL-2 while simultaneously improving targeting efficiency. Low doses of nanodrugs are sufficient to increase the infiltration of natural immune cells (such as NK cells, monocytes, and neutrophils) into tumours, inducing a stronger anti-tumour effect [227]. Moreover, cytokines loaded onto AuNPs not only avoid systemic inflammatory reactions, but their release can also be controlled. For example, Zhang et al. utilised the photothermal properties of AuNPs to regulate the release of cytokine IFN-γ, resulting in approximately 59% cumulative release over 96 h. This approach significantly inhibits postoperative recurrence of lung cancer [228]. Moreover, when combined with IFN-γ, AuNPs exhibit a synergistic effect with ICIs. They not only activate DCs but also generate CD3^+^/CD4^+^ and CD3^+^/CD8^+^ T lymphocytes, reducing the side effects of high therapeutic doses required for a single treatment.

In addition to the above-mentioned cytokines, researchers are investigating relevant agonists, such as TLR7/TLR8 agonists, to stimulate antigen-presenting cells (APCs) and enhance anti-tumour immune responses. Mottas et al. first used AuNPs to deliver a TLR7 immunostimulant (R848) to tumour-draining lymph nodes for the treatment of colon cancer [231]. AuNPs induce DCs maturation and increase the secretion of cytokines (IL-12 and IFN-γ) and chemokines (IL-6 and granulocyte-macrophage colony-stimulating factor) in lymph nodes, enhancing anti-tumour immune responses and improving the effectiveness of immunotherapy. Additionally, AuNPs can directly transport R848 to tumour-draining lymph nodes, thereby preventing drug accumulation and organ damage.

AuNPs can deliver various cytokines to improve the efficacy of cancer immunotherapy. However, cytokines are involved in a series of complex regulatory systems, and the relationship between immune cells and cancer cells is complex. Simultaneously, AuNPs can stimulate immune responses. The combination of proportion and treatment mode will affect the immune system, which means that the final clinical application will face individual differences in therapeutic efficacy.

#### 5.1.2. Cancer Vaccines

Tumour vaccines, which utilise tumour-specific antigens to induce T cell-mediated anti-tumour immunity, are an effective approach for cancer prevention or treatment. Compared with traditional vaccines, vaccines linked with AuNPs can effectively prevent their clearance or degradation by the mononuclear-macrophage system, enhancing the activity of the antigen or antibody [232]. Additionally, AuNPs can target APCs by modifying specific functionalised molecules to enhance antigen cross-presentation, thereby stimulating cytotoxic T lymphocytes (CTLs) to exert anti-tumour effects more effectively [233,234]. Furthermore, AuNPs are capable of easily loading both antigens and adjuvants. Compared with other nanocarrier vectors, their adjuvant properties can enhance the responsiveness of the body to the vaccine. Kevin et al. discovered that AuNPs easily bind to tumour-associated antigens (TAAs) and are encapsulated by functional molecules (such as polysaccharides and lipids) [235]. This not only protects the activity of TAA but also increases the binding dose of the antigen. Compared with free antigen stimulation, the use of polysaccharide-encapsulated AuNPs offers several advantages for improving the immune response of the antigen delivery system. Specifically, AuNPs improve the ability to target APCs expressing dectin-1, thereby enhancing the specificity of the immune response and minimising non-specific immune activation. Additionally, vector-loaded antigens increase the uptake and presentation of antigens by APCs, resulting in mature antibody production and cytokine stimulation of T cells. This suggests that AuNP-loaded antigens are a promising approach for enhancing the immune response to antigens. AuNPs have unique photothermal properties that make them particularly suited for controlling antigen presentation by inducing local thermal disruption of endo/lysosomes [236]. This promotes downstream major histocompatibility complex (MHC)-I antigen cross-presentation, enhancing CTL responses and anticancer immune reactions. Moreover, AuNPs possess adjuvant properties that can heighten the reactivity of antigens in the body. Dykman et al. found that vaccines formulated using AuNPs do not require other adjuvants to stimulate lymphocyte phagocytosis or induce the release of inflammatory mediators [237]. The amount of immune antigen needed to obtain an immune response with this method is much smaller than that with conventional methods. They also discovered that the presence of AuNPs, which bind simultaneously with adjuvants and antigens, results in 5-fold higher antibody titres than the absence of AuNPs [238].

The various advantages of AuNPs make them promising carriers for immunotherapy, especially for constructing tumour vaccines by combining multiple antigens and adjuvants. For instance, AuNPs act as multivalent carriers for loading the MUC1 glycopeptide antigen and α-galactosylceramide immune adjuvant to develop vaccines for treating breast cancer [239]. These vaccines specifically bind to MUC1-positive breast cancer cells. They also produce large numbers of antibodies and kill breast cancer cells via a complement-dependent cytotoxicity-mediated pathway, significantly delaying the growth of breast cancer. Furthermore, GNP-LO91-99, a tumour vaccine prepared using AuNPs as a novel adjuvant and vaccine carrier, has been applied in preclinical studies on advanced melanoma and bladder cancer [240,241]. The GNP-LO91-99 nanovaccine has significant pro-apoptotic and tumour growth inhibitory effects. It induces powerful melanoma-specific CTL responses and reduces tumour immunosuppression by increasing TNF-α and IL-12 levels. Furthermore, the combination of the GNP-LO91-99 nanovaccine and anti-PD-1 or anti-CTLA-4 antibodies can reduce immune checkpoint expression and increase tumour sensitivity to the vaccine. The positive impact of the GNP-LO91-99 nanovaccine in solid tumour immunotherapy is expected to extend to other solid tumours with lymphocyte infiltration, such as non-small cell lung cancer and glioblastoma.

The advantages of AuNPs make them promising carriers for constructing tumour vaccines that combine multiple antigens and adjuvants. Preclinical studies have shown promising results, such as the development of vaccines against breast cancer and advanced melanoma using AuNPs as carriers. These vaccines demonstrate specific binding to cancer cells and inhibit tumour growth via various mechanisms, including the induction of powerful immune responses and reduction in immunosuppression. Although the potential of AuNP-based vaccines has been demonstrated in cellular and animal models, clinical trials have not yet been conducted. This requires comprehensive consideration of the long-term negative effects of AuNPs in organisms. Nonetheless, the long-term stability and modifiability of AuNPs provide them with significant potential for future applications in cancer immunotherapy.

#### 5.1.3. ICIs

The use of AuNPs loaded with ICIs can amplify the anti-tumour T cell immune response while reducing toxicity and adverse events. According to Luo et al., hollow gold nanospheres (HAuNS) have been developed to carry the anti-PD-1 peptide (APP) for regulating drug release. This is particularly effective for reducing the side effects and resistance associated with PD-L1 monotherapy in breast and colorectal cancers [242]. Compared with that with free drugs, HAuNS-APP treatment significantly improves the survival rate of mice. This is evidenced by the significant increase in CD8^+^ T, CD4^+^ T, and immune factors TNF-α, IFN-γ, and IL-2 levels and decrease in the immunosuppressive signalling molecule IL-10 level.

AuNP carriers can protect antibodies, peptides, and oligonucleotides from enzyme degradation, thereby overcoming the weak drug penetration in solid tumours [243]. For instance, Yu et al. developed a biomimetic AuNP platform to protect drugs. This platform carries both anti-PD-L1 peptides and the anti-tumour drug paclitaxel simultaneously, significantly alleviating the immune-inhibitory environment of tumours [221]. This was attributed to the ability of the platform to induce ICD, leading to CD4^+^ T, CD8^+^ T, and NK cell activation and enhanced secretion of immunostimulatory factors (TNF-α and IL-12). Consequently, it achieved a tumour suppression rate of 84.2%. Additionally, combining PD-1 siRNA with AuNPs can enhance the anti-pollution and stability of oligonucleotides. This promotes PD-1 siRNA entry into the nucleus and downregulates PD-1 expression at the gene level. Furthermore, it can increase the production of CD8^+^ T lymphocytes and immune factors (IL-2, TNF-α, and IFN-γ), thereby improving T cell recognition and the killing of tumour cells [114,244]. The multifunctionality of AuNPs enables their integration into diagnosis and therapy. Specifically, Liu et al. first combined AuNPs with PD-L1 siRNA for the simultaneous treatment and diagnosis of melanoma. The nanoplatform not only acts as a vehicle for siRNA delivery to downregulate PD-L1 expression but also a photothermal agent for tumour diagnosis. This nanoplatform has been used for the diagnosis and immunotherapy of lung cancer and melanoma [114,244].

The US Food and Drug Administration (USFDA) has approved the use of ICIs to treat advanced melanoma, renal cell carcinoma, and squamous or non-squamous non-small cell lung cancer [245]. However, the low response rate of cancers to ICIs (usually only 10–30%) remains a challenge [246]. Additionally, monoclonal antibodies used as ICIs may induce autoimmune reactions, resulting in severe immunological adverse events outside the cancer cells. These adverse effects delay medication, introduce uncertainty in drug administration timing and maintenance of dosage, and significantly affect therapeutic outcomes [6,7,247]. Although AuNP technology addresses some issues, further preclinical work is necessary to verify its practical application.

#### 5.1.4. Adoptive Cell Therapy

AuNPs primarily play an auxiliary role in adoptive cell therapy (ACT). ACT involves transferring immune cells that have been expanded or modified in vitro back into cancer patients to boost the anti-tumour immune response [248]. Targeted delivery of IL-12 by AuNPs has been employed to enhance the anti-tumour effect of adaptive T cell therapy on prostate tumours [227]. Low-dose Iso1/Au/IL-12 administration leads to an increase in the number of tumour-infiltrating T cells within the TME while simultaneously reducing the expression of PD-1. This dual effect ultimately improves the recognition and killing abilities of T cells against tumours. AuNPs can enhance T cell immune cytotoxicity by altering T cell culture methods. Using dendrimer-encapsulated AuNPs (Au-DENPs) for CpG non-viral delivery induces the maturation of bone marrow-derived macrophages (BMDMs) for T cell activation [223]. Activated T cells treated with Au-DENPs infiltrate the tumour site, not only killing tumour cells but also triggering adaptive immune responses. This helps maintain the induction of immunotherapy, which is effective in inhibiting tumour metastasis and recurrence.

NK cell therapy has superior therapeutic outcomes and higher safety than T cell therapy [249]. Lin et al. developed a nanoscale immune modulator based on AuNPs to amplify the efficacy of adoptive NK cells in cervical cancer. This immune modulator, named AuNPs@αCD16, is specifically designed to facilitate the infiltration, activation, and killing of NK cells within the TME [250]. The photothermal effect triggered by AuNPs destroys the physical barriers of dense tumour tissues, providing a penetrable TME for NK cell infiltration. Furthermore, AuNPs@αCD16 can activate signalling pathways in tumour cells, leading to an improved release of cytotoxic granules and pro-inflammatory cytokines. Ultimately, this mechanism results in stronger killing effects on solid tumours.

Cytokine-induced killer (CIK) cells are non-MHC-restricted cells that can directly kill tumour cells. Xia et al. combined AuNPs and CD3 antibodies for non-specific binding and tracking the location of CIK cells [226]. This system, combined with PDT and immunotherapy, significantly increases the levels of cytokines, such as IFN-γ, TNF-α, IL-18, and IL-23, and reduces the survival rate of gastric cancer cells by 53%. AuNPs can be used for both therapy and cell tracking, which is lacking for other nanomaterials. Currently, researchers are attempting to apply ACT to solid tumours, and in early studies, it is important to evaluate its systemic response in addition to its efficacy.

#### 5.1.5. Modulation of TME

The TME, which is composed of a complex network of blood vessels and cells, poses a major obstacle to the efficient delivery of drugs and immune cells to the tumour tissue [251,252]. Fortunately, AuNPs have demonstrated the ability to overcome this issue. Specifically, AuNPs have been shown to alter the shape of tumour blood vessels and promote normalisation of the vascular system in melanoma. Furthermore, AuNPs enhance calcium signalling and inhibit Smad2/3 signalling in cancer cells, thereby inducing vascular normalisation. This process indirectly promotes the infiltration of CD3^+^/CD8^+^ T lymphocytes, which ultimately inhibits the growth and metastasis of breast cancer [150]. Additionally, they have been shown to reverse epithelial-mesenchymal transition and reduce the likelihood of lung metastasis [253].

The combination of AuNPs with immune agonists can polarise TAM towards an anti-tumour phenotype, leading to the inhibition of the immunosuppressive environment in the TME [254]. AuNPs can also selectively target M2 macrophages owing to their high specificity for CD163, leading to targeted hyperthermia-induced cell death. This reduces the immunosuppressive effects of the TME and induces the infiltration of T cells, ultimately suppressing the growth of breast cancer [255].

### 5.2. Combinatorial Therapy

Immunotherapy and the combination of PTT, PDT, and RT can modulate the immune environment of tumours. This modulation can enhance immune response and produce synergistic effects in the treatment of tumours. Combination treatment strategies have become increasingly popular owing to their ability to address issues, such as resistance and side effects, associated with immunotherapy. These strategies are widely used not only for in situ tumours but also recurrent and metastatic tumours. By utilising these combination therapies, a more effective and comprehensive approach to tumour treatment can be achieved.

#### 5.2.1. Combined PTT

AuNPs have a high molar extinction coefficient, photobleaching resistance, and remarkable opto-thermal conversion efficiency as photosensitisers, making them suitable for PTT [256]. PTT induces the thermal ablation of cancer cells, leading to the release of TAAs and immune-stimulating molecules [257]. However, repeated long-term PTT increases the expression of inhibitory enzymes and immune checkpoint ligands and recruits immunosuppressive cells, resulting in tumour immune tolerance [258,259]. Combining PTT with immunotherapy stimulates the CD8^+^ T cell-mediated immune response, which inhibits tumour growth and distant metastasis [225]. A recent study has shown that a PTT-immunotherapy strategy based on AuNPs can prevent tumour recurrence [260]. Immunogenic AuNPs synthesised with *Escherichia coli* membrane proteins induced apoptosis and necrosis in tumour cells under 808 nm laser irradiation, resulting in TAA generation and immune activation.

Combining PTT with immunotherapy also has a synergistic effect in the treatment of metastatic tumours [261]. This combination therapy can eradicate both primary tumours and distant metastases while also stimulating an increase in T cell, B cell, and BMDM levels, resulting in effective and durable immunity against tumour recurrence (no tumour growth within 60 days after treatment). AuNPs modified with PD-L1 antibodies synergistically enhance immune response, inhibit in situ tumour growth, and effectively prevent distant tumour metastasis. Based on this, Zhang et al. also developed a highly effective synergistic immunotherapy to detect and inhibit postoperative tumour recurrence [228]. The system kills most tumour cells and exposes a large number of tumour fragments to activate T cells via activated dendritic cells (DCs). CD3^+^/CD8^+^ and CD3^+^/CD4^+^ T lymphocyte levels increased by 44.1% and 34.0%, respectively. Ultimately, activated immune cells secrete immune cytokines that exert anti-tumour effects. This indicates that a sustained immune effect can be generated while killing tumour cells [262].

Generally, the immune response induced by local PTT is weak. However, combination with immunotherapy can significantly enhance immune stimulation and overcome immunosuppression in the TME. In particular, photothermal immunotherapy can effectively treat advanced cancers because it can remove large tumours while enhancing systemic anti-tumour immunity against metastatic tumours.

#### 5.2.2. Combined PDT

PDT is a non-invasive treatment method that utilises the combined effects of photosensitisers and molecular oxygen, triggering apoptosis or cell death, while offering benefits such as localisation and fewer side effects [263,264,265]. Nonetheless, a number of challenges remain, including the limited penetration of light into tissues, poor photostability of photosensitisers, inefficient conversion of light energy, low selectivity for tumour cells, and dependence on strong oxygen [265,266,267]. As enhancers of photosensitisation, AuNPs enhance the sensitising effect of photosensitisers, thereby improving their conversion efficiency and stability. AuNPs have been combined with numerous photosensitisers, such as phthalocyanines [268], porphyrins [269], chlorine 6 [123], and Rose Bengal [270]. Moreover, AuNPs serve as carriers that not only coalesce photosensitisers and oxygen generators to enhance cytotoxic effects but also attach to target receptors (e.g., antibodies and peptides) to improve therapy selectivity [265,271].

AuNPs can act as both photosensitisers and oxygen generator carriers. AuNPs decorated with manganese dioxide cages (AuNCs@MnO_2_, AM) can be used as active oxygen generators in TME. By directly transferring the absorbed light energy to oxygen molecules, they produce sufficient ROS, which can induce ICD [271]. This process induces DC maturation and effector T cell activation, generating an immune response against metastatic triple-negative breast cancer (TNBC), thereby preventing tumour dissemination. However, AM fails to aggregate in the ER, leading to a decreased tumour inhibitory rate. To address this, Li et al. utilised a targeted ER peptide and indocyanine green (ICG)-modified AuNPs to enhance ICD by targeting the ER [272]. Under NIR light irradiation, AuNPs induce ER stress and the exposure of calcium-binding proteins on the cell surface, which serves as an ‘eat-me’ signal to stimulate DCs for antigen presentation [273]. Therefore, AuNPs-ICG activates more CD8^+^ T cells, TNF-α, and IFN-γ, and reduces the number of Tregs. This enhances the immune response and weakens the immunosuppressive TME. These findings suggest that ICD-mediated immunotherapy effectively stimulates the host immune system to exert anticancer effects under specific ER stress.

Although PDT can induce tumour immune responses, its effects are usually too weak to control already-formed tumours [274]. Lin et al. combined AuNPs with anti-CTLA-4 to achieve suppression in subcutaneous xenograft tumour models. The combination not only increased the luminescence intensity and tissue penetration depth of PDT (2-mm increase) but also counteracted the immunosuppressive effect of PDT [275]. The synergistic effect of PDT-induced ICD and PD-L1 blockade can alleviate the immunosuppressive environment of breast cancer. Yu et al. co-loaded a photosensitiser, paclitaxel dimer prodrug, and anti-PD-L1 peptide on AuNPs. The combination effect not only activated CD4^+^ and CD8^+^ T cells and NK cells and promoted the secretion of cytokines (TNF-α and IL-12) but also increased the tumour inhibition rate to 84.2% [221].

Although PDT has shown promising results in cancer immunotherapy, certain issues need to be addressed for further development, such as weak tissue penetration. To address this problem, researchers have recommended enhancing tissue penetration via X-ray-induced PDT, which has considerable potential [276]. Although the combination of AuNPs with this approach has been reported in the literature for cancer treatment, few studies exist regarding its potential for cancer immunotherapy.

#### 5.2.3. Combined RT

RT is a conventional treatment method that primarily utilises ionising radiation to kill cancer cells, and its therapeutic efficacy depends mainly on the radiation sensitivity of the tumour tissue. Owing to their high atomic number and energy absorption coefficient, AuNPs have potential as radiation sensitisers [277,278]. AuNPs absorb radiation energy and initiate a series of ionisation events, such as the production of ROS, mitochondrial and protein damage, and induction of TAA peptides released by cancer cells [11,279].

Research has shown that the synergistic effect of AuNPs and radiation can significantly suppress tumours. Silva et al. used AuNPs as carriers to achieve precise localisation of radioactive isotopes [159,280]. Moreover, the radiosensitising ability of AuNPs can maximise the killing of cancer cells [281,282]. Additionally, AuNPs localised in the cancer cell nucleus can enhance DNA damage and RT-induced immune cell death ICD, increasing the infiltration of macrophages within the tumour [283]. AuNPs can also maximise the destructive effects of photoelectric effects and ROS generation at low radiation energies and induce chemosensitisation. Chen et al. verified this phenomenon in their study, which used AuNP-*Escherichia coli* outer membrane vesicle immune complexes in combination with low-dose radiation to treat glioma cells [284]. The combined treatment led to a significant increase in ROS and TNF-α levels in glioma cells, an 8-fold reduction in tumour volume, and a 35% increase in the survival rate of mice. Even in mice that experienced tumour recurrence after receiving RT, the survival time was extended. However, further experiments are needed to evaluate whether high doses or repeated treatments are helpful in prolonging disease-free time.

AuNPs can promote the deposition of radiation energy in tumours and enhance the immunomodulatory effects of radiation therapy at safe doses [285]. Cao et al. used Au NPs as nanocarriers for CpG ODNs to improve the efficacy of RT/ICIs by mitigating the immunosuppressive microenvironment [124]. AuNP composites repolarise M2 to the M1 phenotype, promoting the secretion of pro-inflammatory factors such as IL-12 and IFN-γ, which leads to an attenuation of the immunosuppressive microenvironment. Furthermore, combined with anti-PD-1, composite-mediated immunomodulation of macrophages can not only activate innate immunity under low-dose X-ray irradiation but also coordinate adaptive immunity. It demonstrated excellent anti-tumour effects on both primary and distant bilateral gliomas. Reduction in the combined toxicity and adverse events of RT and immunotherapy is a particularly important focus of preclinical research. AuNPs can maximise the immune effects of RT while reducing the radiation dosage, thereby reducing the potential toxicity of treatment, and providing a promising platform.

AuNP-based platforms are currently under evaluation in early-phase clinical trials for the treatment of cancer. A particular platform, AuroLase, consisting of a gold-silica nanoshell coated with PEG, has been employed in the PTT of prostate tumours (NCT02680535), lung cancer (NCT01679470), and head and neck cancer (NCT00848042). The efficacy of lesion ablation was confirmed via the systemic injection of gold nanoshell particles in 45 patients with prostate cancer. Notably, the particles were cleared by the liver or isolated from the liver and spleen without adverse effects. These results suggest that gold nanoshell particle injection is a safe and effective method for lesion ablation in patients with prostate cancer. Recently, researchers from the Huntsman Cancer Institute at the University of Utah enrolled 60 patients with prostate cancer to continuously monitor the potential toxicity and efficacy of this protocol (NCT04240639). AuroLase is currently the only inorganic material approved by the USFDA for PTT [286]. The nanoplatform NU-0129 loaded with the oncogenic gene Bcl2Like12 for glioblastoma treatment demonstrated excellent blood–brain barrier ability and no adverse effects in an early phase clinical trial (NCT03020017). In the realm of cancer immunotherapy, there is currently only one early-phase clinical trial based on AuNPs. This clinical trial involved the use of a nanodrug, CYT-6091, that combines recombinant human tumour necrosis factor-alpha (rhTNF) and thiol-PEG with colloidal gold. This drug was tested in phase I clinical trials in eight patients with terminal and metastatic solid cancer (NCT00356980 and NCT00436410), and safety, pharmacokinetics, immunogenicity, and tumour targeting were evaluated. The results indicate that CYT-6091 can selectively deliver therapeutic doses of rhTNF to tumour tissues with greater safety and effectiveness and longer half-life than free rhTNF [287]. Current evidence suggests that AuNPs have great potential for systemic therapeutic effects and exhibit preliminary safety.

Despite the promising results highlighted above, numerous challenges must be addressed before AuNPs can progress to the clinical level. The predominant limiting factor is their safety profile, particularly in terms of their potential interactions with biological systems. Owing to their physical and chemical stability and potential immunogenicity, AuNPs cannot be metabolised by the body during prolonged treatment and can easily accumulate in tissues and organs, leading to toxic effects and systemic immune reactions. The safety of AuNPs is influenced by their size, shape, surface charge, and surface chemistry. Nonetheless, there is contentious debate concerning the biological distribution and toxic mechanisms of AuNPs of various sizes, shapes, charges, formulations, and administration routes, making comparisons challenging. Another limiting factor is the lack of consensus on standardised treatment protocols. Standardising the laser intensity for AuNP-mediated PTT methods remain a challenge owing to the difficulty in comparing the photothermal conversion properties among various types of AuNPs.

## 6. Conclusions and Perspective

This work reviews the design strategies for AuNPs as drug carriers and their applications in cancer immunotherapy. The versatility of AuNPs has been demonstrated by their use as delivery vehicles for a range of drugs, including ICIs, cytotoxic drugs, unstable nucleic acid drugs, and photosensitisers for PDT. Additionally, the light absorption properties of AuNPs make them suitable for PTT and PDT applications. The combination of AuNPs with immunotherapy has been successfully applied for the in situ treatment of various solid tumours, as well as the treatment and prevention of distant metastatic tumours in animal models. Overall, considerable progress has been made in the application of AuNPs in cancer immunotherapy, demonstrating their substantial potential as valuable tools in the treatment of cancer.

Further advances should include the translation of these preclinical technologies into practical clinical applications. Despite a few AuNP-based drugs entering early-stage and phase I clinical trials, their progress is hindered owing to ongoing debates regarding the safety of AuNPs in cellular and animal experiments. Long-term toxicity, aggregation effects, and immune responses in vivo are among the concerns that require thorough investigation. Therefore, studying the interaction between AuNPs and the immune system is crucial for enhancing their clinical application in cancer immunotherapy. It is necessary not only to study whether AuNPs can cause immune reactions, but also to determine the type, duration, and reversibility of immune reactions to provide a basis for clinical selection. Additionally, the combination of PTT and immunotherapy with AuNPs has potential to significantly improve patient survival rates, and ongoing efforts should focus on exploring the efficiency of these combination therapy approaches. Strategies such as preparing various types of AuNPs to increase the absorption of NIR light can increase tissue penetration depth and reduce damage to normal tissue by high-power lasers. It is essential to further explore the effects of AuNP properties on the photothermal conversion effect and develop a universal treatment platform. Future work should prioritise deciphering the interaction between AuNPs and patients, and understanding the biological mechanisms underlying the observed phenomena, rather than solely emphasising the design of complex nanoplatforms.

Establishing clear regulatory solutions and guidelines is crucial for the advancement of AuNP-based nanomedicine. Despite the widespread development and application of nanomedicine, the absence of specific standard protocols and regulatory guidelines has posed challenges for researchers in assessing the toxicity potential of nanomedicines during early-stage testing, resulting in setbacks during later clinical trials. It is of utmost importance for regulatory schemes to continually evolve alongside the applications of nanomedicines, enabling effective clinical work and addressing uncertainties surrounding product definitions, material characterisation, safety assessment, toxicity evaluation, and therapeutic testing. Such measures are vital to ensure the safe and successful translation of AuNP-based nanomedicines into clinical practice.

## Figures and Tables

**Table 1:** Comparison of synthesis methods for AuNPs.

Category	Morphology	Size (nm)	Reducing Agent	Capping Agent	Refs.	Merit	Demerit
Turkevich method	Spherical	15–50	Trisodium citrate		[30]	Homogeneous and dispersed. Uncomplicated and reproducible procedure.	Low yield. Difficult to control shape after >30 nm.
	Spherical	15	Trisodium citrate		[38]		
	Spherical	11.5	Hydroxycarboxylic acid		[39]		
	Spherical	20 ± 2	Sodium citrate		[40]		
	Polyhedron	15–130	Citric acid	TA-PEG	[41]		
Brust–Schiffrin method	Spherical core-shell	32	Sodium tetraborate	Triblock, copolymer	[42]	Thermal and air stability. Size controllable.	Less dispersed. Add harmful agents.
	Cluster	2	NaBH_4_, TOAB	TOAB, 1-hexanethio	[43]		
	Spherical, Triangle	0.24–9	NaBH_4_	Dodecane Thiol, Oleyl amine	[44]		
Seeding-growth method	Hexagonal star	45 ± 7	AA, Trisodium salt	EDTA	[45]	Synthesis of regular Au NRs.	Multiple influence factors. Harmful agent addition.
	Multi Branch	26–50 × 7–10	HEPES, AA	HEPES	[46]		
	Rod	130 × 21	NaBH_4_, AA	CTAB, NaOL	[47]		
	Rod	31.41× 0.96	Sodium citrate, AA	CTAB	[48]		
	Rod	30 × 10	NaBH_4_, AA	CTAB	[49]		
Bio-synthesis method	Spherical	32.2–56.7	Citric acid	Proteins	[50]	Eco-friendly and non-toxic. Biocompatibility. Easy scale-up.	Complicated preparation process. Difficult to control the size and shape. Complex composition analysis.
	Spherical	55–102	Phenolic	Proteins	[51]		
	Spherical Triangle	30 ± 0.25	Biomolecules		[52]		
	Spherical heart-like	15–18	Aromatic primary amines	Amino acids	[53]		
	Triangle, Spherical	10–92	Reductase enzymes	Biomass	[54]		

Abbreviations: TA-PEG: tannic acid-polyethylene glycol; AA: ascorbic acid; TOAB: tetraoctylammonium bromide; CTAB: cetyltrimethylammonium bromide; EDTA: ethylene diamine tetraacetic acid; HEPES: N-2-hydroxyethylpiperazine-N-2-ethane sulfonic acid; NaOL: sodium oleate; Au NRs: gold nanorods.

**Table 2:** List of the surface-functionalized AuNPs loaded with various drugs.

Type of Agent	Surface Functionalization	Connection Method	Refs.
PD-L1 siRNA	poly (sodium 4-styrenesulfonate)	Electrostatic adsorption	[114]
CD11 Ab	/	Van der Waals force	[115]
siRNA	/	Non-covalent bonding	[121]
Glypican-3 siRNA	Polyethyleneimine	Covalent bonding/Au-S	[49]
Trastuzumab	Polyethylene glycol (PEG)	Covalent bonding/Au-S	[122]
Dopamine	PEG	Covalent bonding/Au-S	[123]
CpG-ODNs	Zwitterion 2-methacryloyloxyethyl Phosphorylcholine	Covalent bonding/Au-S	[124]
IDO inhibitor	Polyacrylic acid (PAA)	Covalent bonding/Au-S	[125]
TLR 7 ligands	Thioctic acid	Covalent bonding/Au-S	[126]
OVA	Cysteine residues	Covalent bonding/Au-S	[127]
Anti-VEGF Ab	PEG	Covalent bonding/Au-S	[128]
Anti PD-L1 Ab	11-mercaptoundecanoic acid (MUA)	Covalent bonding/Au-S	[129]

Abbreviations: PD-L1: programmed cell death-ligand 1; CpG-ODNs: CpG oligodeoxynucleotides; IDO: indoleamine-2,3-dioxygenase; TLR 7: toll-like receptor 7; OVA: ovalbumin; VEGF: vascular endothelial growth factor.

**Table 3:** Co-immunotherapy of various diseases is based on AuNPs.

Immunotherapeutic Agents or Gene	Connection Method	Disease	Model	Achievement	Refs.
Anti PD-LI Ab-DOX-HCQ	Covalent bonding/Au-S	Glioma	C6 glioma-bearing mouse	Inhibition of autophagy and TME	[219]
Anti PD-LI Ab-DOX	Covalent bonding/Au-S	Colorectal cancer	CT-26 cells	Increased apoptosis and cell cycle blockage	[130]
TA-PD-I inhibitor	Electrostatic adsorption	Breast cancer Melanoma	4T1-B16F10 mouse B16-C57BL/6 mouse	Improves T cell survival and promotes immune memory	[220]
PXTK-Anti PD-L1 Ab	Electrostatic adsorption	Breast cancer	4T1-BALB/c mouse	Induces ICD and attenuates immunosuppression of the TME	[221]
TKI-Anti PD-L1 Ab	Covalent bonding/Au-S	Acute myeloid leukemia	C1498-C57BL/6 mouse	Induces ferroptosis and promotes T cell immunity	[47]
CpG-ODN	Covalent bonding/Au-S	Breast cancer	4T1 cells	Activates DC and stimulates the secretion of pro-inflammatory cytokines	[222]
CpG	Covalent bonding/Au-S	Melanoma	C57/BL6 mouse	Triggers adaptive immune responses and T cell memory	[223]
TRP-2 peptide-DOX	Electrostatic adsorption	Melanoma	B16F10 tumour-bearing mouse	Increase in cytotoxic CD8^+^ T cell expression	[224]
TLR 3/TLR 9 agonist	/	Colorectal cancer	MC38 tumour-bearing mouse	Combined PTT to destroy TME and eliminate largely (>10 mm^3^) tumour	[225]
CD3 Ab	Covalent bonding/Au-S	Gastric cancer	BALB/c, C57BL/6 nude mouse	Combined PDT to promote tumour suppressor expression	[226]
IL-12	Covalent bonding/Au-S	Fibrosarcoma Breast cancer Prostate cancer	WEHI-164, TS/A C57BL/6 TRAMP mouse	Remodelling the TME, promoting NK cell-mediated anti-tumour effects, and maintaining T cell responses in overt T cell therapy	[227]
IFN-γ	Covalent bonding/Au-S	Lung cancer	C57BL/6 mouse	Detection of tumour recurrence in situ, promotion of T cell immune response	[228]
TGF-β inhibitor	Covalent bonding/Au-S	Melanoma	B16F10 tumour-bearing mouse	Improved specific CD8^+^ T cell cytokine number and function	[229]

Abbreviations: HCQ: hydroxychloroquine; PXTK: paclitaxel dimer prodrug; TKI: tyrosine kinases inhibitor; TRP-2: Tyrosinase-related Protein 2; TGF-β: transforming growth factor-β.

## Data Availability

Not applicable.

## References

[1] Banstola A., Jeong J.H., Yook S. (2020). Immunoadjuvants for cancer immunotherapy: A review of recent developments. Acta Biomater..

[2] Nam J., Son S., Park K.S., Zou W., Shea L.D., Moon J.J. (2019). Cancer nanomedicine for combination cancer immunotherapy. Nat. Rev. Mater..

[3] Mellman I., Coukos G., Dranoff G. (2011). Cancer immunotherapy comes of age. Nature.

[4] Topalian S.L., Drake C.G., Pardoll D.M. (2015). Immune checkpoint blockade: A common denominator approach to cancer therapy. Cancer Cell.

[5] Sharma P., Hu-Lieskovan S., Wargo J.A., Ribas A. (2017). Primary, adaptive, and acquired resistance to cancer immunotherapy. Cell.

[6] Wolchok J.D., Kluger H., Callahan M.K., Postow M.A., Rizvi N.A., Lesokhin A.M., Sznol M. (2013). Nivolumab plus ipilimumab in advanced melanoma. N. Engl. J. Med..

[7] Postow M.A., Sidlow R., Hellmann M.D. (2018). Immune-related adverse events associated with immune checkpoint blockade. N. Engl. J. Med..

[8] Shi J., Kantoff P.W., Wooster R., Farokhzad O.C. (2017). Cancer nanomedicine: Progress, challenges and opportunities. Nat. Rev. Cancer.

[9] Liu Y., Wang X., Shi X., Sun M., Wang L., Hu Z., Zhao C. (2022). A colorimetric sensor for Staphylococcus aureus detection based on controlled click chemical-induced aggregation of gold nanoparticles and immunomagnetic separation. Microchim. Acta.

[10] Sood A., Dev A., Sardoiwala M.N., Choudhury S.R., Chaturvedi S., Mishra A.K., Karmakar S. (2021). Alpha-ketoglutarate decorated iron oxide-gold core-shell nanoparticles for active mitochondrial targeting and radiosensitization enhancement in hepatocellular carcinoma. Mater. Sci. Eng. C.

[11] Her S., Jaffray D.A., Allen C. (2017). Gold nanoparticles for applications in cancer radiotherapy: Mechanisms and recent advancements. Adv. Drug Deliv. Rev..

[12] Bhumkar D.R., Joshi H.M., Sastry M., Pokharkar V.B. (2007). Chitosan reduced gold nanoparticles as novel carriers for transmucosal delivery of insulin. Pharmacol. Res..

[13] Hu X., Zhang Y., Ding T., Liu J., Zhao H. (2020). Multifunctional gold nanoparticles: A novel nanomaterial for various medical applications and biological activities. Front. Bioeng. Biotechnol..

[14] Fang J. (2022). EPR effect-based tumor targeted nanomedicine: A promising approach for controlling cancer. J. Pers. Med..

[15] Riley R.S., June C.H., Langer R., Mitchell M.J. (2019). Delivery technologies for cancer immunotherapy. Nat. Rev. Drug Discov.

[16] Cui T., Liang J.J., Chen H., Geng D.D., Jiao L., Yang J.Y., Ding Y. (2017). Performance of doxorubicin-conjugated gold nanoparticles: Regulation of drug location. ACS Appl. Mater. Interfaces.

[17] Khan A.K., Rashid R., Murtaza G., Zahra A. (2014). Gold nanoparticles: Synthesis and applications in drug delivery. Trop. J. Pharm. Res..

[18] Goddard Z.R., Marín M.J., Russell D.A., Searcey M. (2020). Active targeting of gold nanoparticles as cancer therapeutics. Chem. Soc. Rev..

[19] Amina S.J., Guo B. (2020). A review on the synthesis and functionalization of gold nanoparticles as a drug delivery vehicle. Int. J. Nanomed..

[20] Slovak R., Ludwig J.M., Gettinger S.N., Herbst R.S., Kim H.S. (2017). Immuno-thermal ablations—Boosting the anticancer immune response. J. Immunother. Cancer.

[21] Kroemer G., Galassi C., Zitvogel L., Galluzzi L. (2022). Immunogenic cell stress and death. Nat. Immunol..

[22] Yu X., Pham J.T., Subramani C., Creran B., Yeh Y.-C., Du K., Rotello V.M. (2012). Direct patterning of engineered ionic gold nanoparticles via nanoimprint lithography. Adv. Mater..

[23] Zhang C., Zhang W., Karadas F., Low J., Long R., Liang C., Xiong Y. (2022). Laser-ablation assisted strain engineering of gold nanoparticles for selective electrochemical CO_2_ reduction. Nanoscale.

[24] Xu C., De S., Balu A.M., Ojeda M., Luque R. (2015). Mechanochemical synthesis of advanced nanomaterials for catalytic applications. Chem. Commun..

[25] Ishida Y., Akita I., Sumi T., Matsubara M., Yonezawa T. (2016). Thiolate-protected gold nanoparticles via physical approach: Unusual structural and photophysical characteristics. Sci. Rep..

[26] Sakamoto M., Fujistuka M., Majima T. (2009). Light as a construction tool of metal nanoparticles: Synthesis and mechanism. J. Photochem. Photobiol. C.

[27] Thakkar K.N., Mhatre S.S., Parikh R.Y. (2010). Biological synthesis of metallic nanoparticles. Nanomedicine.

[28] Lee K.X., Shameli K., Yew Y.P., Teow S.Y., Jahangirian H., Rafiee-Moghaddam R., Webster T.J. (2020). Recent developments in the facile bio-synthesis of gold nanoparticles (AuNPs) and their biomedical applications. Int. J. Nanomed..

[29] Khan F.A., Khan F.A. (2020). Synthesis of Nanomaterials: Methods & Technology. Applications of Nanomaterials in Human Health.

[30] Dong J., Carpinone P.L., Pyrgiotakis G., Demokritou P., Moudgil B.M. (2020). Synthesis of precision gold nanoparticles using turkevich method. Kona.

[31] Jayeoye T.J., Eze F.N., Singh S., Olatunde O.O., Benjakul S., Rujiralai T. (2021). Synthesis of gold nanoparticles/polyaniline boronic acid/sodium alginate aqueous nanocomposite based on chemical oxidative polymerization for biological alications. Int. J. Biol. Macromol..

[32] Hutchinson N., Wu Y., Wang Y., Kanungo M., DeBruine A., Kroll E., Zhang W. (2021). Green synthesis of gold nanoparticles using upland cress and their biochemical characterization and assessment. Nanomaterials.

[33] Baig N., Kammakakam I., Falath W. (2021). Nanomaterials: A review of synthesis methods, properties, recent progress, and challenges. Mater. Adv..

[34] Daruich De Souza C., Ribeiro Nogueira B., Rostelato M.E.C.M. (2019). Review of the methodologies used in the synthesis gold nanoparticles by chemical reduction. J. Alloys Compd..

[35] Gupta A., Pandey S., Yadav J.S. (2021). A review on recent trends in green synthesis of gold nanoparticles for tuberculosis. Adv. Pharm. Bull..

[36] Guo S., Wang E. (2007). Synthesis and electrochemical applications of gold nanoparticles. Anal. Chim. Acta.

[37] Yanilkin V.V., Nasretdinova G.N.R., Kokorekin V.A. (2018). Mediated electrochemical synthesis of metal nanoparticles. Russ. Chem. Rev..

[38] Wang J., Mao S., Li H.F., Lin J.M. (2018). Multi-DNAzymes-functionalized gold nanoparticles for ultrasensitive chemiluminescence detection of thrombin on microchip. Anal. Chim. Acta.

[39] Bartosewicz B., Bujno K., Liszewska M., Budner B., Bazarnik P., Płociński T., Jankiewicz B.J. (2018). Effect of citrate substitution by various α-hydroxycarboxylate anions on properties of gold nanoparticles synthesized by Turkevich method. Colloids Surf. A Physicochem. Eng. Asp..

[40] Nebu J., Anjali Devi J.S., Aparna R.S., Aswathy B., Lekha G.M., Sony G. (2018). Fluorescence turn-on detection of fenitrothion using gold nanoparticle quenched fluorescein and its separation using superparamagnetic iron oxide nanoparticle. Sens. Actuators B Chem..

[41] Oh E., Susumu K., Jain V., Kim M., Huston A. (2012). One-pot aqueous phase growth of biocompatible 15–130 nm gold nanoparticles stabilized with bidentate PEG. J. Colloid Interface Sci..

[42] Scaravelli R.C., Dazzi R.L., Giacomelli F.C., Machado G., Giacomelli C., Schmidt V. (2013). Direct synthesis of coated gold nanoparticles mediated by polymers with amino groups. J. Colloid Interface Sci..

[43] Razzaq H., Qureshi R., Cabo-Fernandez L., Schiffrin D.J. (2018). Synthesis of Au clusters-redox centre hybrids by diazonium chemistry employing double layer charged gold nanoparticles. J. Electroanal. Chem..

[44] Xiu-tian-feng E., Zhang Y., Zou J.J., Zhang X., Wang L. (2014). Shape evolution in Brust–Schiffrin synthesis of Au nanoparticles. Mater. Lett..

[45] Huo D., Cao Z., Li J., Xie M., Tao J., Xia Y. (2019). Seed-mediated growth of Au nanospheres into hexagonal stars and the emergence of a hexagonal close-packed phase. Nano Lett..

[46] Lv W., Gu C., Zeng S., Han J., Jiang T., Zhou J. (2018). One-pot synthesis of multi-branch gold nanoparticles and investigation of their SERS performance. Biosensors.

[47] Du Y., Han M., Cao K., Li Q., Pang J., Dou L., Feng S. (2021). Gold nanorods exhibit intrinsic therapeutic activity via controlling N6-methyladenosine-based epitranscriptomics in acute myeloid leukemia. ACS Nano.

[48] Nayef U.M., Khudhair I.M. (2018). Synthesis of gold nanoparticles chemically doped with porous silicon for organic vapor sensor by using photoluminescence. Optik.

[49] Liu Y., Tan M., Fang C., Chen X., Liu H., Feng Y., Min W. (2021). A novel multifunctional gold nanorod-mediated and tumor-targeted gene silencing of GPC-3 synergizes photothermal therapy for liver cancer. Nanotechnology.

[50] Sujitha M.V., Kannan S. (2013). Green synthesis of gold nanoparticles using Citrus fruits (Citrus limon, Citrus reticulata and Citrus sinensis) aqueous extract and its characterization. Spectrochim. Acta A Mol. Biomol. Spectrosc..

[51] Aromal S.A., Vidhu V.K., Philip D. (2012). Green synthesis of well-dispersed gold nanoparticles using Macrotyloma uniflorum. Spectrochim. Acta A Mol. Biomol. Spectrosc..

[52] Venkatesan J., Manivasagan P., Kim S.-K., Kirthi A.V., Marimuthu S., Rahuman A.A. (2014). Marine algae-mediated synthesis of gold nanoparticles using a novel Ecklonia cava. Bioprocess Biosyst. Eng..

[53] Naresh Niranjan D., Ganga Ravindran R., Kannan Badri N., Gurusamy R. (2015). Green chemistry approach for the synthesis of gold nanoparticles using the *Fungus alternaria* sp.. J. Microbiol. Biotechnol..

[54] Shen W., Qu Y., Pei X., Zhang X., Ma Q., Zhang Z., Zhou J. (2016). Green synthesis of gold nanoparticles by a newly isolated strain Trichosporon montevideense for catalytic hydrogenation of nitroaromatics. Biotechnol Lett..

[55] Wuithschick M., Birnbaum A., Witte S., Sztucki M., Vainio U., Pinna N., Polte J. (2015). Turkevich in new robes: Key questions answered for the most common gold nanoparticle synthesis. ACS Nano.

[56] Hamamoto M., Yagyu H. In Two-phase Brust-Schiffrin synthesis of gold nanoparticles dispersion in organic solvent on glass microfluidic device. Proceedings of the 2017 IEEE 17th International Conference on Nanotechnology (IEEE-NANO).

[57] Zhao P., Li N., Astruc D. (2013). State of the art in gold nanoparticle synthesis. Coord. Chem. Rev..

[58] Liu X.Y., Wang J.Q., Ashby C.R., Zeng L., Fan Y.F., Chen Z.S. (2021). Gold nanoparticles: Synthesis, physiochemical properties and therapeutic applications in cancer. Drug Discov. Today.

[59] Turkevich J., Stevenson P.C., Hillier J. (1951). A study of the nucleation and growth processes in the synthesis of colloidal gold. Discuss. Faraday Soc..

[60] Frens G. (1973). Controlled nucleation for the regulation of the particle size in monodisperse gold suspensions. Nat. Phys. Sci..

[61] Polte J. (2015). Fundamental growth principles of colloidal metal nanoparticles—A new perspective. CrystEngComm.

[62] Hammami I., Alabdallah N.M., Jomaa A.A., Kamoun M. (2021). Gold nanoparticles: Synthesis properties and applications. J. King Saud Univ. Sci..

[63] Jakhmola A., Celentano M., Vecchione R., Manikas A., Battista E., Calcagno V., Netti P.A. (2017). Self-assembly of gold nanowire networks into gold foams: Production, ultrastructure and applications. Inorg. Chem. Front..

[64] Jakhmola A., Vecchione R., Onesto V., Gentile F., Profeta M., Battista E., Netti P.A. (2021). A theoretical and experimental study on L-tyrosine and citrate mediated sustainable production of near infrared absorbing twisted gold nanorods. Mater. Sci. Eng. C.

[65] Jakhmola A., Vecchione R., Gentile F., Profeta M., Manikas A.C., Battista E., Netti P.A. (2019). Experimental and theoretical study of biodirected green synthesis of gold nanoflowers. Mater. Today Chem..

[66] Jakhmola A., Vecchione R., Onesto V., Gentile F., Celentano M., Netti P.A. (2021). Experimental and theoretical studies on sustainable synthesis of gold sol displaying dichroic effect. Nanomaterials.

[67] Celentano M., Jakhmola A., Profeta M., Battista E., Guarnieri D., Gentile F., Vecchione R. (2018). Diffusion limited green synthesis of ultra-small gold nanoparticles at room temperature. Colloids Surf. A Physicochem. Eng. Asp..

[68] Jakhmola A., Krishnan S., Onesto V., Gentile F., Profeta M., Manikas A., Netti P.A. (2022). Sustainable synthesis and theoretical studies of polyhedral gold nanoparticles displaying high SERS activity, NIR absorption, and cellular uptake. Mater. Today Chem..

[69] Sengani M., Grumezescu A.M., Rajeswari V.D. (2017). Recent trends and methodologies in gold nanoparticle synthesis—A prospective review on drug delivery aspect. OpenNano.

[70] Brust M., Walker M., Bethell D., Schiffrin D.J., Whyman R. (1994). Synthesis of thiol-derivatised gold nanoparticles in a two-phase Liquid–Liquid system. J. Chem. Soc. Chem. Commun..

[71] Herizchi R., Abbasi E., Milani M., Akbarzadeh A. (2016). Current methods for synthesis of gold nanoparticles. Artif. Cells Nanomed. Biotechnol..

[72] Chen Y., Gu X., Nie C.-G., Jiang Z.-Y., Xie Z.-X., Lin C.-J. (2005). Shape controlled growth of gold nanoparticles by a solution synthesis. ChemComm.

[73] Jana N.R., Gearheart L., Murphy C.J. (2001). Seeding growth for size control of 5−40 nm diameter gold nanoparticles. Langmuir.

[74] Nikoobakht B., El-Sayed M.A. (2003). Preparation and growth mechanism of gold nanorods (NRs) using seed-mediated growth method. Chem. Mater..

[75] Uson L., Sebastian V., Arruebo M., Santamaria J. (2016). Continuous microfluidic synthesis and functionalization of gold nanorods. Chem. Eng. J..

[76] Ward C.J., Tronndorf R., Eustes A.S., Auad M.L., Davis E.W. (2014). Seed-mediated growth of gold nanorods: Limits of length to diameter ratio control. J. Nanomater..

[77] Zhang H., Wang X., Huang K.T., Liang F., Yang Y.W. (2021). Green synthesis of leaning tower[6]arene-mediated gold nanoparticles for label-free detection. Org. Lett..

[78] Alghuthaymi M.A., Rajkuberan C., Santhiya T., Krejcar O., Kuca K., Periakaruppan R., Prabukumar S. (2021). Green synthesis of gold nanoparticles using *Polianthes tuberosa* L. floral extract. Plants.

[79] Mikhailova E.O. (2021). Gold nanoparticles: Biosynthesis and potential of biomedical application. J. Funct. Biomater..

[80] Singh P.K., Kundu S. (2014). Biosynthesis of gold nanoparticles using bacteria. Proc. Natl. Acad. Sci. India Sect. B Biol. Sci..

[81] Gan P.P., Ng S.H., Huang Y., Li S.F. (2012). Green synthesis of gold nanoparticles using palm oil mill effluent (POME): A low-cost and eco-friendly viable approach. Bioresour. Technol..

[82] Cho K.-H., Park J.-E., Osaka T., Park S.-G. (2005). The study of antimicrobial activity and preservative effects of nanosilver ingredient. Electrochimica. Acta.

[83] Deepak P., Amutha V., Kamaraj C., Balasubramani G., Aiswarya D., Perumal P., Shukla A.K., Iravani S. (2019). Chapter 15—Chemical and green synthesis of nanoparticles and their efficacy on cancer cells. Green Synthesis, Characterization and Applications of Nanoparticles.

[84] Gan P.P., Li S.F.Y. (2012). Potential of plant as a biological factory to synthesize gold and silver nanoparticles and their applications. Rev. Environ. Sci. Biotechnol..

[85] Gardea-Torresdey J.L., Tiemann K.J., Gamez G., Dokken K., Tehuacanero S., José-Yacamán M. (1999). Gold nanoparticles obtained by bio-precipitation from gold(III) solutions. J. Nanopart. Res..

[86] Nasaruddin R.R., Chen T., Yao Q., Zang S., Xie J. (2021). Toward greener synthesis of gold nanomaterials: From biological to biomimetic synthesis. Coord. Chem. Rev..

[87] Ghodake G., Lee D.S. (2011). Green synthesis of gold nanostructures using pear extract as effective reducing and coordinating agent. J. Korean Chem. Soc..

[88] Vijayashree I.S., Niranjana P., Prabhu G., Sureshbabu V.V., Manjanna J. (2017). Conjugation of Au nanoparticles with chlorambucil for improved anticancer activity. J. Clust. Sci..

[89] Siddiqi K.S., Husen A. (2017). Recent advances in plant-mediated engineered gold nanoparticles and their application in biological system. J. Trace Elem. Med. Biol..

[90] Teimuri-mofrad R., Hadi R., Tahmasebi B., Farhoudian S., Mehravar M., Nasiri R. (2017). Green synthesis of gold nanoparticles using plant extract: Mini-review. Nano Res..

[91] Mohd Yusof H., Mohamad R., Zaidan U.H., Abdul Rahman N.A. (2019). Microbial synthesis of zinc oxide nanoparticles and their potential application as an antimicrobial agent and a feed supplement in animal industry: A review. J. Anim. Sci. Biotechnol..

[92] Narayanan K.B., Sakthivel N. (2010). Biological synthesis of metal nanoparticles by microbes. Adv. Colloid Interface Sci..

[93] Li J., Li Q., Ma X., Tian B., Li T., Yu J., Hua Y. (2016). Biosynthesis of gold nanoparticles by the extreme bacterium *Deinococcus radiodurans* and an evaluation of their antibacterial properties. Int. J. Nanomed..

[94] Bhambure R., Bule M., Shaligram N., Kamat M., Singhal R. (2009). Extracellular biosynthesis of gold nanoparticles using *Aspergillus niger*—Its characterization and stability. Chem. Eng. Technol..

[95] Wen L., Lin Z., Gu P., Zhou J., Yao B., Chen G., Fu J. (2009). Extracellular biosynthesis of monodispersed gold nanoparticles by a SAM capping route. J. Nanopart. Res..

[96] Siva Kumar K., Kumar G., Prokhorov E., Luna-Bárcenas G., Buitron G., Khanna V.G., Sanchez I.C. (2014). Exploitation of anaerobic enriched mixed bacteria (AEMB) for the silver and gold nanoparticles synthesis. Colloids Surf..

[97] Luo P., Liu Y., Xia Y., Xu H., Xie G. (2014). Aptamer biosensor for sensitive detection of toxin A of Clostridium difficile using gold nanoparticles synthesized by Bacillus stearothermophilus. Biosens. Bioelectron..

[98] Sathiyanarayanan G., Venkatasamy D.V., Saibaba G., Annadurai V., Dineshkumar K., Viswanathan M., Selvin J. (2014). Synthesis of carbohydrate polymer encrusted gold nanoparticles using bacterial exopolysaccharide: A novel and greener approach. RSC Adv..

[99] He S., Guo Z., Zhang Y., Zhang S., Wang J., Gu N. (2007). Biosynthesis of gold nanoparticles using the bacteria Rhodopseudomonas capsulata. Mat Lett..

[100] Feng Y., Yu Y., Wang Y., Lin X. (2007). Biosorption and bioreduction of trivalent aurum by photosynthetic bacteria Rhodobacter capsulatus. Curr. Microbiol..

[101] Zhang X., He X., Wang K., Yang X. (2011). Different active biomolecules involved in biosynthesis of gold nanoparticles by three fungus species. J. Biomed. Nanotechnol..

[102] Kupryashina M.A., Vetchinkina E.P., Burov A.M., Ponomareva E.G., Nikitina V.E. (2013). Biosynthesis of gold nanoparticles by Azospirillum brasilense. Microbiology.

[103] Shankar S.S., Ahmad A., Pasricha R., Sastry M. (2003). Bioreduction of chloroaurate ions by geranium leaves and its endophytic fungus yields gold nanoparticles of different shapes. J. Mater. Chem. A.

[104] Kumar S.A., Abyaneh M.K., Gosavi S.W., Kulkarni S.K., Ahmad A., Khan M.I. (2007). Sulfite reductase-mediated synthesis of gold nanoparticles capped with phytochelatin. Biotechnol. Appl. Biochem..

[105] Qu Y., Pei X., Shen W., Zhang X., Wang J., Zhang Z., Zhou J. (2017). Biosynthesis of gold nanoparticles by Aspergillum sp. WL-Au for degradation of aromatic pollutants. Phys. E Low Dimens. Syst. Nanostruct..

[106] Lee K.D., Nagajyothi P.C., Sreekanth T.V.M., Park S. (2015). Eco-friendly synthesis of gold nanoparticles (AuNPs) using Inonotus obliquus and their antibacterial, antioxidant and cytotoxic activities. J. Ind. Eng. Chem..

[107] Kitching M., Ramani M., Marsili E. (2015). Fungal biosynthesis of gold nanoparticles: Mechanism and scale up. Microb. Biotechnol..

[108] Bai X., Wang Y., Song Z., Feng Y., Chen Y., Zhang D., Feng L. (2020). The Basic Properties of Gold Nanoparticles and their Applications in Tumor Diagnosis and Treatment. Int. J. Mol. Sci..

[109] Yafout M., Ousaid A., Khayati Y., El Otmani I.S. (2021). Gold nanoparticles as a drug delivery system for standard chemotherapeutics: A new lead for targeted pharmacological cancer treatments. Sci. Afr..

[110] Siddique S., Chow J.C.L. (2020). Gold nanoparticles for drug delivery and cancer therapy. Appl. Sci..

[111] Ielo I., Rando G., Giacobello F., Sfameni S., Castellano A., Galletta M., Plutino M.R. (2021). Synthesis, chemical–physical characterization, and biomedical applications of functional gold nanoparticles: A review. Molecules.

[112] Piella J., Bastús N.G., Puntes V. (2017). Size-Dependent Protein–Nanoparticle Interactions in Citrate-Stabilized Gold Nanoparticles: The Emergence of the Protein Corona. Bioconjug. Chem..

[113] Khan M.A.R., Al Mamun M.S., Habib M.A., Islam A.B.M.N., Mahiuddin M., Karim K.M.R., Ara M.H. (2022). A review on gold nanoparticles: Biological synthesis, characterizations, and analytical applications. Results Chem..

[114] Liu B., Cao W., Qiao G., Yao S., Pan S., Wang L., Cui D. (2019). Effects of gold nanoprism-assisted human PD-L1 siRNA on both gene down-regulation and photothermal therapy on lung cancer. Acta Biomater..

[115] Liang R., Xie J., Li J., Wang K., Liu L., Gao Y., Tao J. (2017). Liposomes-coated gold nanocages with antigens and adjuvants targeted delivery to dendritic cells for enhancing antitumor immune response. Biomaterials.

[116] Wang N., Cheng X., Li N., Wang H., Chen H. (2019). Nanocarriers and their loading strategies. Adv. Healthc. Mater..

[117] Park C., Youn H., Kim H., Noh T., Kook Y.H., Oh E.T., Kim C. (2009). Cyclodextrin-covered gold nanoparticles for targeted delivery of an anti-cancer drug. J. Mater. Chem. A.

[118] Veeren A., Ogunyankin M.O., Shin J.E., Zasadzinski J.A. (2022). Liposome-tethered gold nanoparticles triggered by pulsed NIR light for rapid liposome contents release and endosome escape. Pharmaceutics.

[119] Latorre A., Somoza A. (2014). Glutathione-triggered drug release from nanostructures. Curr. Med. Chem..

[120] Yang M., Li J., Gu P., Fan X. (2021). The application of nanoparticles in cancer immunotherapy: Targeting tumor microenvironment. Bioact. Mater..

[121] Poletaeva J., Dovydenko I., Epanchintseva A., Korchagina K., Pyshnyi D., Apartsin E., Pyshnaya I. (2018). Non-covalent associates of sirnas and aunps enveloped with lipid layer and doped with amphiphilic peptide for efficient siRNA delivery. Int. J. Mol. Sci..

[122] Liang S., Sun M., Lu Y., Shi S., Yang Y., Lin Y., Dong C. (2020). Cytokine-induced killer cells-assisted tumor-targeting delivery of Her-2 monoclonal antibody-conjugated gold nanostars with NIR photosensitizer for enhanced therapy of cancer. J. Mater. Chem. B.

[123] Li Z., Yang F., Wu D., Liu Y., Gao Y., Lian H., Zeng L. (2020). Ce6-Conjugated and polydopamine-coated gold nanostars with enhanced photoacoustic imaging and photothermal/photodynamic therapy to inhibit lung metastasis of breast cancer. Nanoscale.

[124] Cao Y., Ding S., Zeng L., Miao J., Wang K., Chen G., Tian G. (2021). Reeducating tumor-associated macrophages using CPG@Au nanocomposites to modulate immunosuppressive microenvironment for improved radio-immunotherapy. ACS Appl. Mater. Interfaces.

[125] Tsao H.-Y., Cheng H.-W., Kuo C.-C., Chen S.-Y. (2022). Dual-Sensitive gold-nanocubes platform with synergistic immunotherapy for inducing immune cycle using NIR-mediated PTT/NO/IDO. Pharmaceuticals.

[126] Shinchi H., Komaki F., Yuki M., Ohara H., Hayakawa N., Wakao M., Suda Y. (2022). Glyco-nanoadjuvants: Impact of linker length for conjugating a synthetic small-molecule tlr7 ligand to glyco-nanoparticles on immunostimulatory effects. ACS Chem. Biol..

[127] Kang S., Ahn S., Lee J., Kim J.Y., Choi M., Gujrati V., Jon S. (2017). Effects of gold nanoparticle-based vaccine size on lymph node delivery and cytotoxic T-lymphocyte responses. J. Control Release.

[128] Silva V., Silva R.N.O., Colli L.G., Carvalho M.H.C., Rodrigues S.F. (2020). Gold nanoparticles carrying or not anti-VEGF antibody do not change glioblastoma multiforme tumor progression in mice. Heliyon.

[129] Wang C., Huang C.H., Gao Z., Shen J., He J., MacLachlan A., Chen P. (2021). Nanoplasmonic Sandwich Immunoassay for Tumor-Derived Exosome Detection and Exosomal PD-L1 Profiling. ACS Sens..

[130] Emami F., Banstola A., Vatanara A., Lee S., Kim J.O., Jeong J.H., Yook S. (2019). Doxorubicin and anti-PD-L1 antibody conjugated gold nanoparticles for colorectal cancer photochemotherapy. Mol. Pharm..

[131] Mbatha L.S., Maiyo F., Daniels A., Singh M. (2021). Dendrimer-coated gold nanoparticles for efficient folate-targeted mRNA delivery in vitro. Pharmaceutics.

[132] Wang S., Song Y., Cao K., Zhang L., Fang X., Chen F., Yan F. (2021). Photothermal therapy mediated by gold nanocages composed of anti-PDL1 and galunisertib for improved synergistic immunotherapy in colorectal cancer. Acta Biomater..

[133] Attia M.F., Anton N., Wallyn J., Omran Z., Vandamme T.F. (2019). An overview of active and passive targeting strategies to improve the nanocarriers efficiency to tumour sites. J. Pharm. Pharmacol..

[134] Byrne J.D., Betancourt T., Brannon-Peppas L. (2008). Active targeting schemes for nanoparticle systems in cancer therapeutics. Adv. Drug Deliv. Rev..

[135] Wu J. (2021). The enhanced permeability and retention (EPR) effect: The significance of the concept and methods to enhance its application. J. Pers. Med..

[136] Matsumura Y., Maeda H. (1986). A new concept for macromolecular therapeutics in cancer chemotherapy: Mechanism of tumoritropic accumulation of proteins and the antitumor agent smancs. Cancer Res..

[137] Islam W., Kimura S., Islam R., Harada A., Ono K., Fang J., Maeda H. (2021). EPR-effect enhancers strongly potentiate tumor-targeted delivery of nanomedicines to advanced cancers: Further extension to enhancement of the therapeutic effect. J. Pers. Med..

[138] Cabral H., Matsumoto Y., Mizuno K., Chen Q., Murakami M., Kimura M., Kataoka K. (2011). Accumulation of sub-100 nm polymeric micelles in poorly permeable tumours depends on size. Nat. Nanotechnol..

[139] Braet F., Wisse E., Bomans P., Frederik P., Geerts W., Koster A., Ringer S. (2007). Contribution of high-resolution correlative imaging techniques in the study of the liver sieve in three-dimensions. Microsc. Res. Tech..

[140] Tan Y., Chen M., Chen H., Wu J., Liu J. (2021). Enhanced ultrasound contrast of renal-clearable luminescent gold nanoparticles. Angew. Chem. Int. Ed..

[141] Arvizo R.R., Miranda O.R., Moyano D.F., Walden C.A., Giri K., Bhattacharya R., Mukherjee P. (2011). Modulating pharmacokinetics, tumor uptake and biodistribution by engineered nanoparticles. PLoS ONE.

[142] Chen L.T., Weiss L. (1973). The role of the sinus wall in the passage of erythrocytes through the spleen. Blood.

[143] Foroozandeh P., Aziz A.A. (2018). Insight into cellular uptake and intracellular trafficking of nanoparticles. Nanoscale Res. Lett..

[144] Ho L.W.C., Liu Y., Han R., Bai Q., Choi C.H.J. (2019). Nano–cell interactions of non-cationic bionanomaterials. Acc. Chem. Res..

[145] Maeda H., Bharate G.Y., Daruwalla J. (2009). Polymeric drugs for efficient tumor-targeted drug delivery based on EPR-effect. Eur. J. Pharm. Biopharm..

[146] Venditto V.J., Szoka F.C. (2013). Cancer nanomedicines: So many papers and so few drugs!. Adv. Drug Deliv. Rev..

[147] Kobayashi H., Watanabe R., Choyke P.L. (2014). Improving conventional enhanced permeability and retention (EPR) effects; what is the appropriate target?. Theranostics.

[148] Danhier F. (2016). To exploit the tumor microenvironment: Since the EPR effect fails in the clinic, what is the future of nanomedicine?. J. Control Release.

[149] Wilhelm S., Tavares A.J., Dai Q., Ohta S., Audet J., Dvorak H.F., Chan W.C.W. (2016). Analysis of nanoparticle delivery to tumours. Nat. Rev. Mater..

[150] Huang N., Liu Y., Fang Y., Zheng S., Wu J., Wang M., Liao W. (2020). Gold Nanoparticles Induce Tumor Vessel Normalization and Impair Metastasis by Inhibiting Endothelial Smad2/3 Signaling. ACS Nano.

[151] Sun X., Zhang G., Keynton R.S., O’Toole M.G., Patel D., Gobin A.M. (2013). Enhanced drug delivery via hyperthermal membrane disruption using targeted gold nanoparticles with PEGylated Protein-G as a cofactor. Nanomedicine.

[152] Taghdisi S.M., Danesh N.M., Lavaee P., Emrani A.S., Hassanabad K.Y., Ramezani M., Abnous K. (2016). Double targeting, controlled release and reversible delivery of daunorubicin to cancer cells by polyvalent aptamers-modified gold nanoparticles. Mater. Sci. Eng. C.

[153] Kim D., Jeong Y.Y., Jon S. (2010). A Drug-loaded aptamer−gold nanoparticle bioconjugate for combined CT imaging and therapy of prostate cancer. ACS Nano.

[154] Yang L., Tseng Y.-T., Suo G., Chen L., Yu J., Chiu W.-J., Lin C.-H. (2015). Photothermal therapeutic response of cancer cells to aptamer–gold nanoparticle-hybridized graphene oxide under NIR Illumination. ACS Appl. Mater. Interfaces.

[155] Han K., Liang Z., Zhou N. (2010). Design strategies for aptamer-based biosensors. Sensors.

[156] Kadkhoda J., Aghanejad A., Safari B., Barar J., Rasta S.H., Davaran S. (2022). Aptamer-conjugated gold nanoparticles for targeted paclitaxel delivery and photothermal therapy in breast cancer. J. Drug Deliv. Sci. Technol..

[157] Dutta B., Barick K.C., Hassan P.A. (2021). Recent advances in active targeting of nanomaterials for anticancer drug delivery. Adv. Colloid Interface Sci..

[158] Tang W., Han L., Lu X., Wang Z., Liu F., Li Y., Ding B. (2021). A Nucleic acid/gold nanorod-based nanoplatform for targeted gene editing and combined tumor therapy. ACS Appl. Mater. Interfaces.

[159] Silva F., D’Onofrio A., Mendes C., Pinto C., Marques A., Campello M.P.C., Paulo A. (2022). Radiolabeled gold nanoseeds decorated with substance p peptides: Synthesis, characterization and in vitro evaluation in glioblastoma cellular models. Int. J. Mol. Sci..

[160] Kim H.S., Lee S.J., Lee D.Y. (2022). Milk protein-shelled gold nanoparticles with gastrointestinally active absorption for aurotherapy to brain tumor. Bioact. Mater..

[161] Obaid G., Chambrier I., Cook M.J., Russell D.A. (2015). Cancer targeting with biomolecules: A comparative study of photodynamic therapy efficacy using antibody or lectin conjugated phthalocyanine-PEG gold nanoparticles. Photochem. Photobiol. Sci..

[162] Tian D., Qin F., Zhao H., Zhang C., Wang H., Liu N., Ai Y. (2021). Bio-Responsive nanoparticle for tumor targeting and enhanced photo-immunotherapy. Colloids Surf. B.

[163] Dings R.P.M., Miller M.C., Griffin R.J., Mayo K.H. (2018). Galectins as molecular targets for therapeutic intervention. Int. J. Mol. Sci..

[164] Jin M., Jin G., Kang L., Chen L., Gao Z., Huang W. (2018). Smart polymeric nanoparticles with pH-responsive and PEG-detachable properties for co-delivering paclitaxel and survivin siRNA to enhance antitumor outcomes. Int. J. Nanomed..

[165] Zhou T., Luo T., Song J., Qu J. (2018). Phasor-fluorescence lifetime imaging microscopy analysis to monitor intercellular drug release from a ph-sensitive polymeric nanocarrier. Anal. Chem..

[166] Lv F., Jin Y., Feng X., Fan M., Ren C., Dai X., Liu H. (2021). Traceable metallic antigen release for enhanced cancer immunotherapy. J. Nanopart. Res.

[167] Asgharzadeh M.R., Barar J., Pourseif M.M., Eskandani M., Jafari Niya M., Mashayekhi M.R., Omidi Y. (2017). Molecular machineries of pH dysregulation in tumor microenvironment: Potential targets for cancer therapy. Bioimpacts.

[168] Khutale G.V., Casey A. (2017). Synthesis and characterization of a multifunctional gold-doxorubicin nanoparticle system for pH triggered intracellular anticancer drug release. Eur. J. Pharm. Biopharm..

[169] Xu W., Qian J., Hou G., Suo A., Wang Y., Wang J., Yao Y. (2017). Hyaluronic acid-functionalized gold nanorods with ph/nir dual-responsive drug release for synergetic targeted photothermal chemotherapy of breast cancer. ACS Appl. Mater. Interfaces.

[170] Ma K., Cheng Y., Wei X., Chen D., Zhao X., Jia P. (2021). Gold embedded chitosan nanoparticles with cell membrane mimetic polymer coating for pH-sensitive controlled drug release and cellular fluorescence imaging. J. Biomater. Appl..

[171] Suarasan S., Focsan M., Potara M., Soritau O., Florea A., Maniu D., Astilean S. (2016). Doxorubicin-incorporated nanotherapeutic delivery system based on gelatin-coated gold nanoparticles: Formulation, drug release, and multimodal imaging of cellular internalization. ACS Appl. Mater Interfaces.

[172] Chen G., Xie Y., Peltier R., Lei H., Wang P., Chen J., Sun H. (2016). Peptide-decorated gold nanoparticles as functional nano-capping agent of mesoporous silica container for targeting drug delivery. ACS Appl. Mater. Interfaces.

[173] Tan Y., Liu L., Wang Y., Liu J. (2018). pH-Regulated Surface Plasmon Absorption from Ultrasmall Luminescent Gold Nanoparticles. Adv. Opt. Mater..

[174] You Y., Xu Z., Chen Y. (2018). Doxorubicin conjugated with a trastuzumab epitope and an MMP-2 sensitive peptide linker for the treatment of HER2-positive breast cancer. Drug Deliv..

[175] Liu X., Su Q., Song H., Shi X., Zhang Y., Zhang C., Wang W. (2021). PolyTLR7/8a-conjugated, antigen-trapping gold nanorods elicit anticancer immunity against abscopal tumors by photothermal therapy-induced in situ vaccination. Biomaterials.

[176] Witzel I., Marx A.K., Müller V., Wikman H., Matschke J., Schumacher U., Oliveira-Ferrer L. (2017). Role of HYAL1 expression in primary breast cancer in the formation of brain metastases. Breast Cancer Res. Treat..

[177] Cheng D., Ji Y., Wang B., Wang Y., Tang Y., Fu Y., Zhu W. (2021). Dual-responsive nanohybrid based on degradable silica-coated gold nanorods for triple-combination therapy for breast cancer. Acta Biomater..

[178] Kang T.Y., Park K., Kwon S.H., Chae W.-S. (2020). Surface-engineered nanoporous gold nanoparticles for light-triggered drug release. Op. Mater..

[179] Pourjavadi A., Bagherifard M., Doroudian M. (2020). Synthesis of micelles based on chitosan functionalized with gold nanorods as a light sensitive drug delivery vehicle. J. Biol. Macromol..

[180] Han D., Xu J., Wang Z., Yang N., Li X., Qian Y., Xu S. (2018). Penetrating effect of high-intensity infrared laser pulses through body tissue. RSC Adv..

[181] Park J.-E., Kim M., Hwang J.-H., Nam J.-M. (2017). Golden Opportunities: Plasmonic Gold Nanostructures for Biomedical Applications based on the Second Near-Infrared Window. Small Methods.

[182] Shakeri-Zadeh A., Zareyi H., Sheervalilou R., Laurent S., Ghaznavi H., Samadian H. (2021). Gold nanoparticle-mediated bubbles in cancer nanotechnology. J. Control Release.

[183] Lyu Z., Zhou F., Liu Q., Xue H., Yu Q., Chen H. (2016). A Universal platform for macromolecular deliveryinto cells using gold nanoparticle layers via the photoporation effect. Adv. Funct. Mater..

[184] Hasanzadeh Kafshgari M., Agiotis L., Largilliere I., Patskovsky S., Meunier M. (2021). Antibody-Functionalized Gold Nanostar-Mediated On-Resonance Picosecond Laser Optoporation for Targeted Delivery of RNA Therapeutics. Small.

[185] Wayteck L., Xiong R., Braeckmans K., De Smedt S.C., Raemdonck K. (2017). Comparing photoporation and nucleofection for delivery of small interfering RNA to cytotoxic T cells. J. Control. Release.

[186] Sauvage F., Nguyen V.P., Li Y., Harizaj A., Sebag J., Roels D., De Smedt S.C. (2022). Laser-induced nanobubbles safely ablate vitreous opacities in vivo. Nat. Nanotechnol..

[187] Yao C., Rudnitzki F., He Y., Zhang Z., Huttmann G., Rahmanzadeh R. (2020). Cancer cell-specific protein delivery by optoporation with laser-irradiated gold nanorods. J. Biophotonics.

[188] Song X., Zhu W., Ge X., Li R., Li S., Chen X., Yang H. (2021). A New class of NIR-II gold nanocluster-based protein biolabels for in vivo tumor-targeted imaging. Angew. Chem. Int. Ed. Engl..

[189] Bibikova O., Singh P., Popov A., Akchurin G., Skaptsov A., Skovorodkin I., Tuchin V. (2017). Shape-dependent interaction of gold nanoparticles with cultured cells at laser exposure. Laser Phys. Lett..

[190] Pylaev T.E., Efremov Y., Avdeeva E.S., Antoshin A.A., Shpichka A.I., Khlebnikova T.M., Khlebtsov N.G. (2021). Optoporation and recovery of living cells under Au nanoparticle layer-mediated NIR-laser irradiation. ACS Appl. Nano Mater..

[191] Shanei A., Sazgarnia A. (2019). An overview of therapeutic applications of ultrasound based on synergetic effects with gold nanoparticles and laser excitation. Iran J. Basic Med. Sci..

[192] Hornsby T., Jakhmola A., Kolios M.C., Tavakkoli J.J. Significance of non-thermal effects in LIPUS induced drug release from gold nanoparticle drug carriers. Proceedings of the 2021 IEEE UFFC Latin America Ultrasonics Symposium (LAUS).

[193] Jakhmola A., Hornsby T., Rod K., Tavakkoli J. A Novel Gold Nanoparticles Drug Delivery System: Design and ex vivo Tissue Testing. Proceedings of the 2020 IEEE International Ultrasonics Symposium (IUS).

[194] Lin L., Fan Y., Gao F., Jin L., Li D., Sun W., Du L. (2018). UTMD-promoted co-delivery of gemcitabine and mir-21 inhibitor by dendrimer-entrapped gold nanoparticles for pancreatic cancer therapy. Theranostics.

[195] Chan T.G., Morse S.V., Copping M.J., Choi J.J., Vilar R. (2018). Targeted Delivery of DNA-Au Nanoparticles across the Blood-Brain Barrier Using Focused Ultrasound. ChemMedChem.

[196] Chen R., Shi J., Zhu B., Zhang L., Cao S. (2020). Mesoporous hollow hydroxyapatite capped with smart polymer for multi-stimuli remotely controlled drug delivery. Microporous Mesoporous Mater..

[197] Chen D.S., Mellman I. (2013). Oncology Meets Immunology: The Cancer-Immunity Cycle. Immunity.

[198] Rivera Vargas T., Apetoh L. (2017). Danger signals: Chemotherapy enhancers?. Immunol. Rev..

[199] Dykman L.A., Khlebtsov N.G. (2017). Immunological properties of gold nanoparticles. Chem. Sci..

[200] Mohammapdour R., Ghandehari H. (2022). Mechanisms of immune response to inorganic nanoparticles and their degradation products. Adv. Drug Deliv. Rev..

[201] Zhu M., Du L., Zhao R., Wang H.Y., Zhao Y., Nie G., Wang R.F. (2020). Cell-penetrating nanoparticles activate the inflammasome to enhance antibody production by targeting microtubule-associated protein 1-light chain 3 for degradation. ACS Nano.

[202] Dey A.K., Gonon A., Pecheur E.I., Pezet M., Villiers C., Marche P.N. (2021). Impact of gold nanoparticles on the functions of macrophages and dendritic cells. Cells.

[203] Ibrahim K.E., Bakhiet A.O., Awadalla M.E., Khan H.A. (2018). A priming dose protects against gold nanoparticles-induced proinflammatory cytokines mRNA expression in mice. Nanomedicine.

[204] Malaczewska J. (2015). The splenocyte proliferative response and cytokine secretion in mice after oral administration of commercial gold nanocolloid. Pol. J. Vet. Sci..

[205] Nicolas-Boluda A., Laurent G., Bazzi R., Roux S., Donnadieu E., Gazeau F. (2021). Two step promotion of a hot tumor immune environment by gold decorated iron oxide nanoflowers and light-triggered mild hyperthermia. Nanoscale.

[206] Duan X., Chan C., Lin W. (2019). Nanoparticle-mediated immunogenic cell death enables and potentiates cancer immunotherapy. Angew. Chem. Int. Ed. Engl..

[207] Zitvogel L., Kepp O., Senovilla L., Menger L., Chaput N., Kroemer G. (2010). Immunogenic tumor cell death for optimal anticancer therapy: The calreticulin exposure pathway. Clin. Cancer Res..

[208] Martins I., Wang Y., Michaud M., Ma Y., Sukkurwala A.Q., Shen S., Kroemer G. (2014). Molecular mechanisms of ATP secretion during immunogenic cell death. Cell Death Differ..

[209] Panaretakis T., Joza N., Modjtahedi N., Tesniere A., Vitale I., Durchschlag M., Kroemer G. (2008). The co-translocation of ERp57 and calreticulin determines the immunogenicity of cell death. Cell Death Differ..

[210] Krysko D.V., Garg A.D., Kaczmarek A., Krysko O., Agostinis P., Vandenabeele P. (2012). Immunogenic cell death and DAMPs in cancer therapy. Nat Rev. Cancer.

[211] Agostinis P., Berg K., Cengel K.A., Foster T.H., Girotti A.W., Gollnick S.O., Golab J. (2011). Photodynamic therapy of cancer: An update. CA Cancer J. Clin..

[212] Hwang H.S., Shin H., Han J., Na K. (2018). Combination of photodynamic therapy (PDT) and anti-tumor immunity in cancer therapy. J. Pharm Investig.

[213] Zhou J., Wang G., Chen Y., Wang H., Hua Y., Cai Z. (2019). Immunogenic cell death in cancer therapy: Present and emerging inducers. J. Cell Mol. Med..

[214] Granja A., Pinheiro M., Sousa C.T., Reis S. (2021). Gold nanostructures as mediators of hyperthermia therapies in breast cancer. Biochem. Pharmacol..

[215] Niikura K., Matsunaga T., Suzuki T., Kobayashi S., Yamaguchi H., Orba Y., Sawa H. (2013). Gold nanoparticles as a vaccine platform: Influence of size and shape on immunological responses in vitro and in vivo. ACS Nano.

[216] Yen H.J., Hsu S.H., Tsai C.L. (2009). Cytotoxicity and immunological response of gold and silver nanoparticles of different sizes. Small.

[217] Kroemer G., Zitvogel L. (2018). The breakthrough of the microbiota. Nat. Rev. Immunol..

[218] Zhang Y., Zhang Z. (2020). The history and advances in cancer immunotherapy: Understanding the characteristics of tumor-infiltrating immune cells and their therapeutic implications. Cell Mol. Immunol..

[219] Ruan S., Xie R., Qin L., Yu M., Xiao W., Hu C., Gao H. (2019). Aggregable nanoparticles-enabled chemotherapy and autophagy inhibition combined with anti-PD-L1 antibody for improved glioma treatment. Nano Lett..

[220] Luo L., Li X., Zhang J., Zhu C., Jiang M., Luo Z., You J. (2021). Enhanced immune memory through a constant photothermal-metabolism regulation for cancer prevention and treatment. Biomaterials.

[221] Yu W., He X., Yang Z., Yang X., Xiao W., Liu R., Gao H. (2019). Sequentially responsive biomimetic nanoparticles with optimal size in combination with checkpoint blockade for cascade synergetic treatment of breast cancer and lung metastasis. Biomaterials.

[222] Ong C., Cha B.G., Kim J. (2019). Mesoporous silica nanoparticles doped with gold nanoparticles for combined cancer immunotherapy and photothermal therapy. ACS Appl. Bio Mater..

[223] Chen H., Fan Y., Hao X., Yang C., Peng Y., Guo R., Cao X. (2020). Adoptive cellular immunotherapy of tumors via effective CpG delivery to dendritic cells using dendrimer-entrapped gold nanoparticles as a gene vector. J. Mater. Chem. B.

[224] Won J.E., Wi T.I., Lee C.M., Lee J.H., Kang T.H., Lee J.W., Han H.D. (2021). NIR irradiation-controlled drug release utilizing injectable hydrogels containing gold-labeled liposomes for the treatment of melanoma cancer. Acta Biomater..

[225] Nam J., Son S., Park K.S., Moon J.J. (2021). Photothermal therapy combined with neoantigen cancer vaccination for effective immunotherapy against large established tumors and distant metastasis. Adv. Ther..

[226] Xia F., Hou W., Liu Y., Wang W., Han Y., Yang M., Cui D. (2018). Cytokine induced killer cells-assisted delivery of chlorin e6 mediated self-assembled gold nanoclusters to tumors for imaging and immuno-photodynamic therapy. Biomaterials.

[227] Gasparri A.M., Sacchi A., Basso V., Cortesi F., Freschi M., Rrapaj E., Curnis F. (2019). Boosting interleukin-12 antitumor activity and synergism with immunotherapy by targeted delivery with isoDGR-tagged nanogold. Small.

[228] Zhang Y., Wang T., Tian Y., Zhang C., Ge K., Zhang J., Wang H. (2021). Gold nanorods-mediated efficient synergistic immunotherapy for detection and inhibition of postoperative tumor recurrence. Acta Pharm. Sin. B.

[229] Yang Y.S., Moynihan K.D., Bekdemir A., Dichwalkar T.M., Noh M.M., Watson N., Irvine D.J. (2018). Targeting small molecule drugs to T cells with antibody-directed cell-penetrating gold nanoparticles. Biomater. Sci..

[230] Zhao Z., Zheng L., Chen W., Weng W., Song J., Ji J. (2019). Delivery strategies of cancer immunotherapy: Recent advances and future perspectives. J. Hematol. Oncol..

[231] Mottas I., Bekdemir A., Cereghetti A., Spagnuolo L., Yang Y.S., Muller M., Bourquin C. (2019). Amphiphilic nanoparticle delivery enhances the anticancer efficacy of a TLR7 ligand via local immune activation. Biomaterials.

[232] Carabineiro S.A.C. (2017). Applications of gold nanoparticles in nanomedicine: Recent advances in vaccines. Molecules.

[233] Popescu C.R., Grumezescu M.A. (2015). Metal based frameworks for drug delivery systems. Curr. Top. Med. Chem..

[234] Yin H., Kanasty R.L., Eltoukhy A.A., Vegas A.J., Dorkin J.R., Anderson D.G. (2014). Non-viral vectors for gene-based therapy. Nat. Rev. Genet..

[235] Trabbic K.R., Kleski K.A., Barchi J.J. (2021). A Stable gold nanoparticle-based vaccine for the targeted delivery of tumor-associated glycopeptide antigens. ACS Bio. Med. Chem. Au.

[236] Cao F., Yan M., Liu Y., Liu L., Ma G. (2018). Photothermally controlled MHC class I restricted CD8(+) T-cell responses elicited by hyaluronic acid decorated gold nanoparticles as a vaccine for cancer immunotherapy. Adv. Healthc. Mater..

[237] Dykman L.A., Staroverov S.A., Bogatyrev V.A., Shchyogolev S.Y. (2010). Adjuvant properties of gold nanoparticles. Nanotechnol. Russ..

[238] Dykman L.A., Staroverov S.A., Fomin A.S., Khanadeev V.A., Khlebtsov B.N., Bogatyrev V.A. (2018). Gold nanoparticles as an adjuvant: Influence of size, shape, and technique of combination with CpG on antibody production. Int. Immunopharmacol..

[239] Liu Y., Wang Z., Yu F., Li M., Zhu H., Wang K., Zhao W. (2021). The Adjuvant of α-galactosylceramide presented by gold nanoparticles enhances antitumor immune responses of MUC1 antigen-based tumor vaccines. Int. J. Nanomed..

[240] Teran-Navarro H., Calderon-Gonzalez R., Salcines-Cuevas D., Garcia I., Marradi M., Freire J., Alvarez-Dominguez C. (2019). Pre-clinical development of Listeria-based nanovaccines as immunotherapies for solid tumours: Insights from melanoma. Oncoimmunology.

[241] Teran-Navarro H., Zeoli A., Salines-Cuevas D., Marradi M., Montoya N., Gonzalez-Lopez E., Alvarez-Dominguez C. (2022). Gold Glyconanoparticles Combined with 91–99 Peptide of the Bacterial Toxin, Listeriolysin O, Are Efficient Immunotherapies in Experimental Bladder Tumors. Cancers.

[242] Luo L., Yang J., Zhu C., Jiang M., Guo X., Li W., You J. (2018). Sustained release of anti-PD-1 peptide for perdurable immunotherapy together with photothermal ablation against primary and distant tumors. J. Control. Release.

[243] Ruan S., Xiao W., Hu C., Zhang H., Rao J., Wang S., Gao H. (2017). Ligand-mediated and enzyme-directed precise targeting and retention for the enhanced treatment of glioblastoma. ACS Appl. Mater. Interfaces.

[244] Gao Y., Ouyang Z., Yang C., Song C., Jiang C., Song S., Shi X. (2021). Overcoming T cell exhaustion via immune checkpoint modulation with a dendrimer-based hybrid nanocomplex. Adv. Healthc. Mater..

[245] Hodi F.S., O’Day S.J., McDermott D.F., Weber R.W., Sosman J.A., Haanen J.B., Urba W.J. (2010). Improved survival with ipilimumab in patients with metastatic melanoma. N. Engl. J. Med..

[246] Herbst R.S., Soria J.-C., Kowanetz M., Fine G.D., Hamid O., Gordon M.S., Hodi F.S. (2014). Predictive correlates of response to the anti-PD-L1 antibody MPDL3280A in cancer patients. Nature.

[247] Friedman C.F., Proverbs-Singh T.A., Postow M.A. (2016). Treatment of the immune-related adverse effects of immune checkpoint inhibitors: A review. JAMA Oncol..

[248] Rohaan M.W., Wilgenhof S., Haanen J.B.A.G. (2019). Adoptive cellular therapies: The current landscape. Virchows Arch..

[249] Bald T., Krummel M.F., Smyth M.J., Barry K.C. (2020). The NK cell–cancer cycle: Advances and new challenges in NK cell–based immunotherapies. Nat. Immunol..

[250] Lin X., Li F., Gu Q., Wang X., Zheng Y., Li J., Liu X. (2022). Gold-seaurchin based immunomodulator enabling photothermal intervention and αCD16 transfection to boost NK cell adoptive immunotherapy. Acta Biomater..

[251] Murciano-Goroff Y.R., Warner A.B., Wolchok J.D. (2020). The future of cancer immunotherapy: Microenvironment-targeting combinations. Cell Res..

[252] Iwadate Y. (2016). Epithelial-mesenchymal transition in glioblastoma progression. Oncol. Lett..

[253] Li W., Li X., Liu S., Yang W., Pan F., Yang X.Y., Pan Y. (2017). Gold nanoparticles attenuate metastasis by tumor vasculature normalization and epithelial-mesenchymal transition inhibition. Int. J. Nanomed..

[254] Zhang S., Xie F., Li K., Zhang H., Yin Y., Yu Y., Gao J. (2022). Gold nanoparticle-directed autophagy intervention for antitumor immunotherapy via inhibiting tumor-associated macrophage M2 polarization. Acta Pharm. Sin. B.

[255] Wang B., Huang Y. (2022). Antitumor effects of targeted killing of tumor-associated macrophages under photothermal conditions. Lasers Med. Sci..

[256] Odion R., Liu Y., Vo-Dinh T. (2021). Plasmonic Gold nanostar-mediated photothermal immunotherapy. IEEE J. Sel Top Quantum Electron..

[257] Evans S.S., Repasky E.A., Fisher D.T. (2015). Fever and the thermal regulation of immunity: The immune system feels the heat. Nat. Rev. Immunol..

[258] Peng J., Xiao Y., Li W., Yang Q., Tan L., Jia Y., Qian Z. (2018). Photosensitizer micelles together with ido inhibitor enhance cancer photothermal therapy and immunotherapy. Adv. Sci..

[259] Toraya-Brown S., Sheen M.R., Zhang P., Chen L., Baird J.R., Demidenko E., Fiering S. (2014). Local hyperthermia treatment of tumors induces CD8(+) T cell-mediated resistance against distal and secondary tumors. Nanomedicine.

[260] Hwang J., An E.-K., Kim S.-J., Zhang W., Jin J.-O. (2022). *Escherichia coli* mimetic gold nanorod-mediated photo- and immunotherapy for treating cancer and its metastasis. ACS Nano.

[261] Liu Y., Maccarini P., Palmer G.M., Etienne W., Zhao Y., Lee C.-T., Vo-Dinh T. (2017). Synergistic immuno photothermal nanotherapy (SYMPHONY) for the treatment of unresectable and metastatic cancers. Sci. Rep..

[262] Zhang Z., Liu S., Zhang B., Qiao L., Zhang Y., Zhang Y. (2020). T cell dysfunction and exhaustion in cancer. Front. Cell Dev. Biol..

[263] Chatterjee D.K., Fong L.S., Zhang Y. (2008). Nanoparticles in photodynamic therapy: An emerging paradigm. Adv. Drug Deliv. Rev..

[264] Youssef Z., Jouan-Hureaux V., Colombeau L., Arnoux P., Moussaron A., Baros F., Frochot C. (2018). Titania and silica nanoparticles coupled to Chlorin e6 for anti-cancer photodynamic therapy. Photodiagn. Photodyn. Ther..

[265] Dhaini B., Kenzhebayeva B., Ben-Mihoub A., Gries M., Acherar S., Baros F., Frochot C. (2021). Peptide-conjugated nanoparticles for targeted photodynamic therapy. Nanophotonics.

[266] Youssef Z., Arnoux P., Colombeau L., Moussaron A., Toufaily J., Hamieh T., Roques-Carmes T. (2017). Two approaches for elaborating sensitized TiO2 nanoparticles of potential effect in photodynamic therapy. Photodiagn. Photodyn. Ther..

[267] He J.S., Liu S.J., Zhang Y.R., Chu X.D., Lin Z.B., Zhao Z., Pan J.H. (2021). The application of and strategy for gold nanoparticles in cancer immunotherapy. Front Pharmacol..

[268] Obaid G., Chambrier I., Cook M.J., Russell D.A. (2012). Targeting the oncofetal thomsen–friedenreich disaccharide using jacalin-peg phthalocyanine gold nanoparticles for photodynamic cancer therapy. Angew. Chem. Int. Ed..

[269] Zeng J., Yang W., Shi D., Li X., Zhang H., Chen M. (2018). Correction to “Porphyrin derivative conjugated with gold nanoparticles for dual-modality photodynamic and photothermal therapies in vitro”. ACS Biomater. Sci. Eng..

[270] Fadel M., Fadeel D.A., Tawfik A., El-Kholy A.I., Mosaad Y.O. (2022). Rose Bengal-gold-polypyrrole nanoparticles as a photothermal/photodynamic dual treatment of recalcitrant plantar warts: Animal and clinical study. J. Drug Deliv. Sci. Technol..

[271] Liang R., Liu L., He H., Chen Z., Han Z., Luo Z., Cai L. (2018). Oxygen-boosted immunogenic photodynamic therapy with gold nanocages@manganese dioxide to inhibit tumor growth and metastases. Biomaterials.

[272] Li W., Yang J., Luo L., Jiang M., Qin B., Yin H., You J. (2019). Targeting photodynamic and photothermal therapy to the endoplasmic reticulum enhances immunogenic cancer cell death. Nat. Commun..

[273] Li Y., Liu X., Zhang X., Pan W., Li N., Tang B. (2021). Immunogenic cell death inducers for enhanced cancer immunotherapy. Chem. Commun..

[274] Castano A.P., Mroz P., Wu M.X., Hamblin M.R. (2008). Photodynamic therapy plus low-dose cyclophosphamide generates antitumor immunity in a mouse model. PNAS.

[275] Lin B., Liu J., Wang Y., Yang F., Huang L., Lv R. (2020). Enhanced upconversion luminescence-guided synergistic antitumor therapy based on photodynamic therapy and immune checkpoint blockade. Chem. Mater..

[276] Youssef Z., Vanderesse R., Colombeau L., Baros F., Roques-Carmes T., Frochot C., Gazzali A.M. (2017). The application of titanium dioxide, zinc oxide, fullerene, and graphene nanoparticles in photodynamic therapy. Cancer Nanotechnol..

[277] Hainfeld J.F., Dilmanian F.A., Slatkin D.N., Smilowitz H.M. (2008). Radiotherapy enhancement with gold nanoparticles. J. Pharm. Pharmacol..

[278] Shiryaeva E.S., Baranova I.A., Sanochkina E.V., Dement’eva O.V., Kartseva M.E., Shishmakova E.M., Feldman V.I. (2022). On the mechanism of radiation sensitization by gold nanoparticles under X-ray irradiation of oxygen-free aqueous organic solutions: A spin trapping study. Radiat. Phys. Chem..

[279] Dimitriou N.M., Tsekenis G., Balanikas E.C., Pavlopoulou A., Mitsiogianni M., Mantso T., Georgakilas A.G. (2017). Gold nanoparticles, radiations and the immune system: Current insights into the physical mechanisms and the biological interactions of this new alliance towards cancer therapy. Pharmacol.Ther..

[280] Silva F., Paulo A., Pallier A., Même S., Tóth É., Gano L., Cabral Campello M.P. (2020). Dual imaging gold nanoplatforms for targeted radiotheranostics. Materials.

[281] Yao X., Huang C., Chen X., Yi Z., Sanche L. (2015). Chemical Radiosensitivity of DNA Induced by Gold Nanoparticles. J. Biomed Nanotechnol..

[282] Rosa S., Connolly C., Schettino G., Butterworth K.T., Prise K.M. (2017). Biological mechanisms of gold nanoparticle radiosensitization. Cancer Nanotechnol..

[283] Janic B., Brown S.L., Neff R., Liu F., Mao G., Chen Y., Wen N. (2021). Therapeutic enhancement of radiation and immunomodulation by gold nanoparticles in triple negative breast cancer. Cancer Biol. Ther..

[284] Chen M.H., Liu T.Y., Chen Y.C., Chen M.H. (2021). Combining augmented radiotherapy and immunotherapy through a nano-gold and bacterial outer-membrane vesicle complex for the treatment of glioblastoma. Nanomaterials.

[285] Xie J., Gong L., Zhu S., Yong Y., Gu Z., Zhao Y. (2019). Emerging strategies of nanomaterial-mediated tumor radiosensitization. Adv. Mater..

[286] Sung D., Sanchez A., Tward J.D. (2023). Successful salvage brachytherapy after infusion of gold auroshell nanoshells for localized prostate cancer in a human patient. Adv. Radiat..

[287] Libutti S.K., Paciotti G.F., Byrnes A.A., Alexander H.R., Gannon W.E., Walker M., Tamarkin L. (2010). Phase I and pharmacokinetic studies of CYT-6091, a novel PEGylated colloidal gold-rhTNF nanomedicine. Clin. Cancer Res..

